# Supplementary material for: Resource availability, utilisation and cost in the provision of critical care in Tanzania: a protocol for a systematic review
Source: BMJ Open. 2021 Aug 25;11(8):e050881. doi: 10.1136/bmjopen-2021-050881 (PMC8388301; doi:10.1136/bmjopen-2021-050881)
Supplement: Supplementary data [file bmjopen-2021-050881supp001.pdf]

Appendix 1: Search Strategies

1) Medline search strategy

	Interface: Ovid MEDLINE<br>Date of Search:<br>Number of hits:
**Number**	**Search**
1	(((Critical care or emergency care or critical emergency care or essential emergency) and critical care) or intensive care or early goal directed therapy or neonatal or acute care or emergency medicine or trauma or emergency medical services or ICU or Peri-operative care or maternal emergencies or cardiovascular support or inotropic support or renal support).mp.
2	(General ward care or general inpatient care or hospital care or Oxygen therapy or artificial ventilation or mechanical ventilation or Non-invasive ventilation or Continuous Positive Airway Pressure or High flow nasal oxygen or HFO or HFNO).mp.
3	(Critical illness or sick children or acute paediatrics or emergency obstetric care or poly-trauma or Severe illness or Life-threatening illness or Acute illness or HIV or Malaria or TB or sepsis or trauma or burns or pneumonia or emergency surgery or shock or haemorrhage or respiratory failure or coma or unconsciousness or meningitis or choking or anuria or acute kidney injury).mp.
4	(Cost or expen or spending or invest- or Financ or financial burden or financial impact or financial consequence or economic or economic burden or economic impact or economic consequence or Direct cost or indirect cost or medical cost or non-medical cost or nonmedical cost or opportunity cost or Resource or resource or resource utilisation or resource utilization or health service utilisation or health service utilization or Provider cost or health system cost or hospital cost or system cost or provider cost or hospital cost or societal cost or insurance or reimburse- or cost of illness or cost analysis or economic modelling).mp.
5	Tanzania.mp. or exp Tanzania/
6	1 or 2 or 3 or 4
7	5 and 6
8	limit 7 to yr="2010 -Current"

2) Embase search strategy

	Interface: Ovid Embase<br>Date of Search:

	Number of hits:
**Number**	**Search**
1	(((Critical care or emergency care or critical emergency care or essential emergency) and critical care) or intensive care or early goal directed therapy or neonatal or acute care or emergency medicine or trauma or emergency medical services or ICU or Peri-operative care or maternal emergencies or cardiovascular support or inotropic support or renal support).mp.
2	(General ward care or general inpatient care or hospital care or Oxygen therapy or artificial ventilation or mechanical ventilation or Non-invasive ventilation or Continuous Positive Airway Pressure or High flow nasal oxygen or HFO or HFNO).mp.
3	(Critical illness or sick children or acute paediatrics or emergency obstetric care or poly-trauma or Severe illness or Life-threatening illness or Acute illness or HIV or Malaria or TB or sepsis or trauma or burns or pneumonia or emergency surgery or shock or haemorrhage or respiratory failure or coma or unconsciousness or meningitis or choking or anuria or acute kidney injury).mp.
4	(Cost or expen or spending or invest- or Financ or financial burden or financial impact or financial consequence or economic or economic burden or economic impact or economic consequence or Direct cost or indirect cost or medical cost or non-medical cost or nonmedical cost or opportunity cost or Resource or resource or resource utilisation or resource utilization or health service utilisation or health service utilization or Provider cost or health system cost or hospital cost or system cost or provider cost or hospital cost or societal cost or insurance or reimburse- or cost of illness or cost analysis or economic modelling).mp.
5	Tanzania.mp. or exp Tanzania/
6	1 or 2 or 3 or 4
7	5 and 6
8	limit 7 to yr="2010 -Current"

3) Global health Search Strategy

	Interface: Ovid Global Health<br>Date of Search:<br>Number of hits:
**Number**	**Search**
1	(((Critical care or emergency care or critical emergency care or essential emergency) and critical care) or intensive care or early goal directed therapy or neonatal or acute care or emergency medicine or trauma or emergency medical services or ICU or Peri-operative care or maternal emergencies or cardiovascular support or inotropic support or renal support).mp.

2	(General ward care or general inpatient care or hospital care or Oxygen therapy or artificial ventilation or mechanical ventilation or Non-invasive ventilation or Continuous Positive Airway Pressure or High flow nasal oxygen or HFO or HFNO).mp.
3	(Critical illness or sick children or acute paediatrics or emergency obstetric care or poly-trauma or Severe illness or Life-threatening illness or Acute illness or HIV or Malaria or TB or sepsis or trauma or burns or pneumonia or emergency surgery or shock or haemorrhage or respiratory failure or coma or unconsciousness or meningitis or choking or anuria or acute kidney injury).mp.
4	(Cost or expen or spending or invest- or Financ or financial burden or financial impact or financial consequence or economic or economic burden or economic impact or economic consequence or Direct cost or indirect cost or medical cost or non-medical cost or nonmedical cost or opportunity cost or Resource or resource or resource utilisation or resource utilization or health service utilisation or health service utilization or Provider cost or health system cost or hospital cost or system cost or provider cost or hospital cost or societal cost or insurance or reimburse- or cost of illness or cost analysis or economic modelling).mp.
5	Tanzania.mp. or exp Tanzania/
6	1 or 2 or 3 or 4
7	5 and 6
8	limit 7 to yr="2010 -Current"
