# Supplementary material for: Resource availability, utilisation and cost in the provision of critical care in Tanzania: a protocol for a systematic review
Source: BMJ Open. 2021 Aug 25;11(8):e050881. doi: 10.1136/bmjopen-2021-050881 (PMC8388301; doi:10.1136/bmjopen-2021-050881)
Supplement: Supplementary data [file bmjopen-2021-050881supp002.pdf]

Appendix 2: Inclusion and exclusion criteria

Inclusion criterion

For a study to be included it should fulfil the following criterion

- Published in English in a peer reviewed journal
- Reports forms or types of critical care offered, critical care services offered and or costs and resources used in the provision of care in Tanzania
- Includes costs from a provider perspective
- Articles published from 2010

Exclusion criterion

Studies will be excluded in case of the following;

- Published in any other languages except English
- Studies carried out outside Tanzania
- Studies with costs estimated from patient perspective
- Studies without full text versions
- Studies published before 2010
