# Supplementary material for: Resource availability, utilisation and cost in the provision of critical care in Tanzania: a protocol for a systematic review
Source: BMJ Open. 2021 Aug 25;11(8):e050881. doi: 10.1136/bmjopen-2021-050881 (PMC8388301; doi:10.1136/bmjopen-2021-050881)
Supplement: Supplementary data [file bmjopen-2021-050881supp003.pdf]

Synthesis Without Meta-analysis (SWiM) reporting items

SWiM is intended to complement and be used as an extension to PRISMA			
**SWiM reporting item**	**Item description**	**Page in manuscript where item is reported**	**Other***
Methods			
**1** Grouping studies for synthesis	The studies will be grouped into two; studies that report cost and studies that report on forms of critical care. For the studies that report costs of critical care, the synthesis will be by level of health facility/hospital, health services offered, type of costs reported, data sources, study designs, geographical location (rural and urban). The forms of critical care will be synthesized by level of health facility. The rationale for this analysis is that cost and forms of critical care vary between the subgroups mentioned above.	Page 7	
**2** Describe the standardised metric and transformation methods used	The costs will be presented in two currencies that is, the USD and TZS. All extracted costs will be adjusted to the 2019 value of USD and TZS using the World Bank GDP deflators. The GDP deflator method is explained by Chowdhury[1].	Page 7	
**3** Describe the synthesis methods	Collection and reported of cost data are usually heterogenous making it difficult to have reliable and representative findings after pooling the reported costs from different papers. In order to synthesize results in this study, we plan to categorize costs according to source of the costs, type of costs reported and costs per services offered. This will give us a summative understanding of the results.<br><br>For forms of critical care, we are planning to synthesize this data by categorizing it by level of health facility/hospital because different levels of health facilities have different amount of resources to deliver critical care thereby delivering varied critical care services.	Page 7	
**4** Criteria used to prioritise	The criteria used to prioritise results will be based on the objectives that this study sets up to achieve which rotate around forms of critical care and cost of critical care which includes resources	Page 5 and Appendix 1	

Kazibwe J, *et al. BMJ Open* 2021; 11:e050881. doi: 10.1136/bmjopen-2021-050881

results for summary and synthesis	used. Studies will be included in this study using the inclusion and exclusion criteria available in the appendix.		
**5** Investigation of heterogeneity in reported effects	Heterogeneity of the costs will be investigated using the appraisal checklist.	Page 7	
**6** Certainty of evidence	In order to assess certainty of evidence, we plan to use the Reference Case for Estimating the Costs of Global Health Services and Interventions[25] to identify likely bias in the reported findings of included studies. In addition, for studies that do not report costs, we will use the Newcastle Ottawa Scale to assess bias. Any study that scores less than 5 will be excluded.	Page 7	
**7** Data presentation methods	We will use tables to describe the study characteristics and costs. The table of characteristics will contain the details of studies like author, year of publication, study design, intervention, health facility level among others). The costs will be presented in different tables by category of the cost e.g equipment, services, human resources, diagnosis.<br><br>In addition, the forms of critical care will be presented using a figure showing the different formats of critical care at the different levels of the health system.	Page 7	
Results			
**8** Reporting results	N/A (This will be reported on after the data extraction is done)	N/A	
Discussion			
**9** Limitations of the synthesis	N/A (This will be responded to after the synthesis is done)	N/A	

Kazibwe J, *et al. BMJ Open* 2021; 11:e050881. doi: 10.1136/bmjopen-2021-050881

Source: Campbell M, McKenzie JE, Sowden A, Katikireddi SV, Brennan SE, Ellis S, Hartmann-Boyce J, Ryan R, Shepperd S, Thomas J, Welch V, Thomson H. Synthesis without meta-analysis (SWiM) in systematic reviews: reporting guideline BMJ 2020;368:l6890 http://dx.doi.org/10.1136/bmj.l6890
