# Supplementary material for: Resource availability, utilisation and cost in the provision of critical care in Tanzania: a protocol for a systematic review
Source: BMJ Open. 2021 Aug 25;11(8):e050881. doi: 10.1136/bmjopen-2021-050881 (PMC8388301; doi:10.1136/bmjopen-2021-050881)
Supplement: Supplementary data [file bmjopen-2021-050881supp004.pdf]

PRISMA CHECKLIST

Title of study: Resource availability, utilization and cost in the provision of critical care in Tanzania: A protocol for a systematic review

Item Number	Item	Description	Page and line number
1	Title	Identify the report as a systematic review	Page 1, lines 4-5
2	Abstract	Summary of the study	Page 2, Lines 39-61
3	Rationale	Describe the rationale for the review in the context of existing knowledge	Page 3, Lines 118-135
4	Objectives	Provide an explicit statement of the objective(s) or question(s) the review addresses	Page 4, Lines 137-146
5	Eligibility criteria	Specify the inclusion and exclusion criteria for the review and how studies were grouped for the syntheses	Page 6, Lines 205-209 And appendix 2
6	Information sources	Specify all databases, registers, websites, organisations, reference lists, and other sources searched or consulted to identify studies.	Page 5, Lines 175-187
7	Search strategy	Present the full search strategies for all databases, registers, and websites, including any filters and limits used	Page 5, Lines 175-199 And Appendix 1
8	Selection process	Specify the methods used to decide whether a study met the inclusion criteria of the review, including how many reviewers screened each record and each report retrieved, whether they worked independently, and, if applicable, details of automation tools used in the process	Page 7, Lines 212-217
9	Data collection process	Specify the methods used to collect data from reports, including how many reviewers collected data from each report, whether they worked independently, any processes for obtaining or confirming data from study investigators, and, if applicable, details of automation tools used in the process	Page 7, Lines 219-224
10a	Data items	List and define all outcomes for which data were sought. Specify whether all results that were compatible with each outcome domain in each study were sought (for example, for all measures, time	Table 1: Page 4

		points, analyses), and, if not, the methods used to decide which results to collect	
10b	Data items	List and define all other variables for which data were sought (such as participant and intervention characteristics, funding sources). Describe any assumptions made about any missing or unclear information	Page 7, Lines 219-222
11	Study risk of bias	Specify the methods used to assess risk of bias in the included studies, including details of the tool(s) used, how many reviewers assessed each study and whether they worked independently, and, if applicable, details of automation tools used in the process	Page 7, Lines 225-233
12	Effect measures	Specify for each outcome the effect measure(s) (such as risk ratio, mean difference) used in the synthesis or presentation of results	Not applicable
13a	Synthesis methods	Describe the processes used to decide which studies were eligible for each synthesis (such as tabulating the study intervention characteristics and comparing against the planned groups for each synthesis (item #5))	Page 7, Lines 222-227
13b	Synthesis methods	Describe any methods required to prepare the data for presentation or synthesis, such as handling of missing summary statistics or data conversions	Page 7, Lines 234-243
13c	Synthesis methods	Describe any methods used to tabulate or visually display results of individual studies and syntheses	Page 7, Lines 235-242
13d	Synthesis methods	Describe any methods used to synthesise results and provide a rationale for the choice(s). If meta-analysis was performed, describe the model(s), method(s) to identify the presence and extent of statistical heterogeneity, and software package(s) used	Page 7, Lines 235-242
13e	Synthesis methods	Describe any methods used to explore possible causes of heterogeneity among study results (such as subgroup analysis, meta-regression)	Not applicable
13f	Synthesis methods	Describe any sensitivity analyses conducted to assess robustness of the synthesised results	Not applicable
14	Reporting bias assessment	Describe any methods used to assess risk of bias due to missing results in a synthesis (arising from reporting biases)	Not applicable at this stage

15	Certainty assessment	Describe any methods used to assess certainty (or confidence) in the body of evidence for an outcome	Page 7, 237-238
16a	Study selection	Describe the results of the search and selection process, from the number of records identified in the search to the number of studies included in the review, ideally using a flow diagram	Not applicable at this stage
16b	Study selection	Cite studies that might appear to meet the inclusion criteria, but which were excluded, and explain why they were excluded	Not applicable at this stage
17	Study characteristics	Cite each included study and present its characteristics	Not applicable at this stage
18	Risk of bias in studies	Present assessments of risk of bias for each included study	Not applicable at this stage
19	Results of individual studies	For all outcomes, present for each study (a) summary statistics for each group (where appropriate) and (b) an effect estimate and its precision (such as confidence/credible interval), ideally using structured tables or plots	Not applicable at this stage
20a	Results of syntheses	For each synthesis, briefly summarise the characteristics and risk of bias among contributing studies	Not applicable at this stage
20b	Results of syntheses	Present results of all statistical syntheses conducted. If meta-analysis was done, present for each the summary estimate and its precision (such as confidence/credible interval) and measures of statistical heterogeneity. If comparing groups, describe the direction of the effect	Not applicable at this stage
20c	Results of syntheses	Present results of all investigations of possible causes of heterogeneity among study results	Not applicable at this stage
20d	Results of syntheses	Present results of all sensitivity analyses conducted to assess the robustness of the synthesised results	Not applicable at this stage
21	Risk of reporting bias in syntheses	Present assessments of risk of bias due to missing results (arising from reporting biases) for each synthesis assessed	Not applicable at this stage
22	Certainty of evidence	Present assessments of certainty (or confidence) in the body of evidence for each outcome assessed	Not applicable at this stage

23a	Discussion	Provide a general interpretation of the results in the context of other evidence	Not applicable at this stage
23b	Discussion	Discuss any limitations of the evidence included in the review	Not applicable at this stage
23c	Discussion	Discuss any limitations of the review processes used	Not applicable at this stage
23d	Discussion	Discuss implications of the results for practice, policy, and future research	Not applicable at this stage
24a	Registration and protocol	Provide registration information for the review, including register name and registration number, or state that the review was not registered	Page 4, Lines 150-151
24b	Registration and protocol	Indicate where the review protocol can be accessed, or state that a protocol was not prepared	Page 4, Lines 150-151
24c	Registration and protocol	Describe and explain any amendments to information provided at registration or in the protocol	Not applicable at this stage
25	Support	Describe sources of financial or non-financial support for the review, and the role of the funders or sponsors in the review	Page 10, lines 360-361
26	Competing interests	Declare any competing interests of review authors	Page 10, Lines 362-364
27	Availability of data, code and other materials	Report which of the following are publicly available and where they can be found: template data collection forms; data extracted from included studies; data used for all analyses; analytic code; any other materials used in the review	Not applicable at this stage
