# Supplementary material for: Resource availability, utilisation and cost in the provision of critical care in Tanzania: a protocol for a systematic review
Source: BMJ Open. 2021 Aug 25;11(8):e050881. doi: 10.1136/bmjopen-2021-050881 (PMC8388301; doi:10.1136/bmjopen-2021-050881)
Supplement: Author's manuscript [file bmjopen-2021-050881.draft_revisions.pdf]

BMJ Open is committed to open peer review. As part of this commitment we make the peer review history of every article we publish publicly available.

When an article is published we post the peer reviewers' comments and the authors' responses online. We also post the versions of the paper that were used during peer review. These are the versions that the peer review comments apply to.

The versions of the paper that follow are the versions that were submitted during the peer review process. They are not the versions of record or the final published versions. They should not be cited or distributed as the published version of this manuscript.

BMJ Open is an open access journal and the full, final, typeset and author-corrected version of record of the manuscript is available on our site with no access controls, subscription charges or pay-per-view fees (http://bmjopen.bmj.com).

If you have any questions on BMJ Open's open peer review process please email info.bmjopen@bmj.com

BMJ Open

Resource availability, utilization and cost in the provision of critical care in Tanzania: A protocol for a systematic review

Journal:	*BMJ Open*
Manuscript ID	bmjopen-2021-050881
Article Type:	Protocol
Date Submitted by the Author:	03-Mar-2021
Complete List of Authors:	Kazibwe, Joseph; Imperial College London, Department of Infectious Disease Epidemiology<br>Shah, Hiral A.; Imperial College London, Department of Infectious Disease Epidemiology; Center for Global Development, Department of Infectious Disease Epidemiology<br>Kuwawenaruwa, A; Ifakara Health Institute<br>Schell, Carl Otto; Karolinska Institutet, Department of Global Public Health; Uppsala University, Department of Global Public Health<br>Khalid, Karima; Ifakara Health Institute; Muhimbili University of Health and Allied Sciences<br>Tran, Phuong Bich; University of Antwerp, Department of Family and Population Health<br>Ghosh, Srobana; Center for Global Development<br>Baker, Tim; Karolinska Institutet, Department of Global Public Health<br>GUINNESS, LORNA; Center for Global Development; London School of Hygiene and Tropical Medicine, GLOBAL HEALTH & DEVELOPMENT
Keywords:	INTENSIVE & CRITICAL CARE, HEALTH ECONOMICS, Health policy < HEALTH SERVICES ADMINISTRATION & MANAGEMENT

SCHOLARONE™
Manuscripts

I, the Submitting Author has the right to grant and does grant on behalf of all authors of the Work (as defined in the below author licence), an exclusive licence and/or a non-exclusive licence for contributions from authors who are: i) UK Crown employees; ii) where BMJ has agreed a CC-BY licence shall apply, and/or iii) in accordance with the terms applicable for US Federal Government officers or employees acting as part of their official duties; on a worldwide, perpetual, irrevocable, royalty-free basis to BMJ Publishing Group Ltd ("BMJ") its licensees and where the relevant Journal is co-owned by BMJ to the co-owners of the Journal, to publish the Work in this journal and any other BMJ products and to exploit all rights, as set out in our licence.

The Submitting Author accepts and understands that any supply made under these terms is made by BMJ to the Submitting Author unless you are acting as an employee on behalf of your employer or a postgraduate student of an affiliated institution which is paying any applicable article publishing charge ("APC") for Open Access articles. Where the Submitting Author wishes to make the Work available on an Open Access basis (and intends to pay the relevant APC), the terms of reuse of such Open Access shall be governed by a Creative Commons licence – details of these licences and which Creative Commons licence will apply to this Work are set out in our licence referred to above.

Other than as permitted in any relevant BMJ Author's Self Archiving Policies, I confirm this Work has not been accepted for publication elsewhere, is not being considered for publication elsewhere and does not duplicate material already published. I confirm all authors consent to publication of this Work and authorise the granting of this licence.

For peer review only

Resource availability, utilization and cost in the provision of critical care in Tanzania: A protocol for a systematic review

Corresponding Author

Joseph Kazibwe
Department of Infectious Disease Epidemiology
Imperial College London, London
Norfolk Place, London W2 1PG – UK
j.kazibwe@imperial.ac.uk
+447561876477

Authors and affiliation

Joseph Kazibwe[1], Hiral A. Shah[1,5], August Kuwawenaruwa[2], Carl Otto Schell[7,8], Karima Khalid[2,3], Phuong Bich Tran[4], Srobana Ghosh[5], Tim Baker[2,6], Lorna Guinness[5]

1. Department of Infectious Disease Epidemiology, Imperial College London, London – UK
2. Ifakara Health Institute, Dar es Salaam – Tanzania
3. Muhimbili University of Health and Allied Sciences, Dar es Salaam – Tanzania
4. Department of Family and Population Health (FAMPOP), University of Antwerp, Antwerp - Belgium
5. Center for Global Development (CGD), London – UK
6. Department of Clinical Research, London School of Hygiene and Tropical Medicine, UK
7. Department of Global Public Health, Karolinska Institutet, Stockholm, Sweden
8. Centre for Clinical Research Sörmland, Uppsala University, Eskilstuna, Sweden

Word count (excluding title page, abstract, references, figures and tables): 1,773

Key words: Critical care, costing, utilization of resources in critical care, forms of critical care, critical illness, low resource settings

For peer review only

Abstract

Introduction

Critical care is essential in saving lives of those that are critically ill, however, provision of critical care can be costly and heterogenous across lower resource settings. This paper describes the protocol for a systematic review of the literature that aims to identify the reported costs and resources available for the provision of critical care and the forms of critical care provision in Tanzania.

Methods and analysis

The review will follow the Preferred Reporting Items for Systematic Reviews and Meta-Analyses (PRISMA) guidelines. Three databases (MEDLINE, Embase and Global Health) will be searched to identify articles that report the forms of critical care, resources used in the provision of critical care in Tanzania, their availability and the associated costs. The search strategy will be developed from four key concepts; critical care provision, critical illness, resource use, Tanzania. The articles that fulfil the inclusion and exclusion criteria will be assessed for quality using the Reference Case for Estimating the Costs of Global Health Services and Interventions checklist. The extracted data will be summarized using descriptive statistics including frequencies, mean and median of the quantity and costs of resources used in the components of critical care services, depending on the data availability.

Ethics and dissemination

This study is a review of secondary data, and is part of the Provision Of Essential Treatment In Critical Illness in the context of the COVID-19 pandemic (POETIC). Ethical clearance was sought and granted from the Tanzanian National Institute of Medical Research (reference: NIMR/HQ/R.8a/Vol. IX/3537) and London School of Hygiene and Tropical Medicine (LSHTM ethics ref: 22866). We will publish the review in a peer reviewed journal as an open access article in addition to presenting the findings at conferences and public scientific gatherings.

Strengths and weaknesses of the proposed study

- This is the first systematic review of the literature around the costs and resources used in critical care and forms of critical care in a lower resource setting such as Tanzania.
- The search strategy broadly and comprehensively includes studies on critical care and costs of inpatient care in Tanzania increasing chances of including all published studies on the subject.
- This study has a fully developed population, intervention, comparison and outcome (PICO) framework.
- This study will provide an overview on the current evidence base on resources and costs for decision-making in critical care in Tanzania and other low resource settings.
- The study will include only articles published in English.

For peer review only

Background

The COVID19 pandemic is now synonymous with being a critical care crisis with both high income and low-and-middle income countries (LMICs) struggling to meet demand for critical care services.[1,2] This has led to the care of critical illness becoming an urgent issue and a point of focus in global health with stakeholders providing recommendations on what interventions should be scaled up, while knowledge on the potential costs involved remains sparse[1,3,4].

Critical care entails the care given to a patient in need of specialist monitoring, treatment and attention, for example for a patient with a life-threatening illness or injury[5]. Over the years, there have been great advances in the provision of critical care for example invasive and non-invasive monitoring techniques, mechanical ventilation (MV), and renal replacement therapy, among others which have resulted in reduced mortality rates over time among patients with critical illness especially in high income countries[6].

Despite the advances made in the provision of critical care in LMICs[7–9], the limited availability of intensive care units (ICUs) and the low numbers of trained personnel in such settings persist. In addition, the frequent power cuts and inadequate supply of oxygen in some areas make advanced care challenging. The equipment in an ICU can be expensive and may not be affordable or available in many LMICs with an already constrained healthcare budget[10,11]. In a global mapping of ICU bed capacity, Egypt had the highest ICU bed capacity in Africa at 10.6 per 100,000 which compares poorly with 59.5 per 100,000 in Monaco which had the highest ICU bed capacity globally[12]. Even where ICUs are available in LMICs settings, the services offered are expensive, making them inaccessible and unaffordable for the majority of critically ill patients. This limited availability of critical care services in LMICs has been linked to the high ICU related deaths of between 30%-80% of all ICU admissions[8]. In fact, most critically ill patients are cared for in general hospital wards[13,14].

Given the constraints that LMICs face when having to provide critical care, recent research has proposed the concept of Essential Emergency and Critical Care (EECC) which focuses on providing low-cost care for critical illness in any place within the hospital[1,9]. EECC consists of effective life-saving actions of low cost and complexity, is appropriate for all hospital settings and for all critically ill patients irrespective of their age, sex, location or underlying medical condition[9]. EECC has been proposed as a key component in universal health coverage (UHC). Research is ongoing into the policy and economic aspects of EECC in Tanzania and Kenya to inform EECC implementation in these and other low resourced health system settings.

To inform this work, it is necessary to review the existing knowledge about the current resource availability, utilization and cost in the provision of critical care in Tanzania. Several studies have been conducted to estimate the resource use and cost of critical care globally but the majority of these estimates are confined to high income countries and may not be generalizable to the LMICs settings [15–17]. Even studies that are reported as international estimates for example Negrini et al that looked at the resource use and cost of critical care in Germany, Hungary, UK and France[18]. In addition, the majority of studies focus on the costs incurred in an ICU only[17] which may not reflect the resources used for all critically illness, especially in LMICs. Here, we describe the protocol of a systematic review that will identify the existing evidence on the quantification of current resources available and the economic costs in the provision of critical care in Tanzania.

For peer review only

Aim

This paper describes the protocol for a systematic review of the literature that aims to identify the reported costs and resources available for the provision of critical care and the forms of critical care provision in Tanzania.

Research questions

1. What are forms of critical care provided in the health system in Tanzania?
2. What resources are reported to be available and have been utilised in the provision of critical care in Tanzania?
3. What are the reported costs of available resources (including oxygen supplies) used in critical care provision in Tanzania?

Methods

The systematic review will follow the Preferred Reporting Items for Systematic Reviews and Meta-Analyses (PRISMA) guidelines[19] and the protocol was registered with PROSPERO; registration number: CRD42020221923.

The review will use a PICO (Population, Intervention, Comparator, Outcome) framework to enable a comprehensive and unbiased search of existing literature and identify the key search terms[20] (see table 1) . Critical care as a concept does not have an internationally accepted definition. The review defines critical care as the components of care that support a critically ill patient's vital organ functions where critical illness is a state of ill health with vital organ dysfunction, a high risk of imminent death if action is not taken and the potential for reversibility. Critical care is ideally team-based and multi-professional and can be given in any location. Critical care can be high-intensity e.g mechanical ventilation, requiring highly specialised services, or it can be low intensity resuscitative and supportive care e.g oxygen provided using a face mask.

Table 1: PICO (Population, Intervention, Comparator, Outcome) Framework used to inform the search strategy.

**Element**	**Description**
Population	Any patient in need of critical care
Intervention	Inpatient services that could form part of critical care provision
Comparison	No critical care
Outcomes	Critical care services available<br>Cost of the different inputs to critical care provision per patient<br>Quantity and type of resources used in provision of critical care per patient

For peer review only

Search strategy

Electronic databases (Medline (Ovid), Embase (embase.com) and Global Health) will be systematically searched for articles published between 2010 to present. A ten-year time frame will be considered to ensure that findings reflect the present-day resource utilization paradigm in critical care provision as resources used and their costs vary with the nature of technology and clinical guidelines in place. Bibliographies of included articles will be reviewed to find relevant articles that fulfil the inclusion criteria but not yet identified. Authors whose abstracts will be considered for inclusion, but the full text is not accessible online, will be contacted and requested for article access. Google and Google Scholar will also be used to search for published articles that may not have been indexed within the databases. As internet search engines typically return several thousand results, the searches will be restricted to the first fifty hits and links to potentially relevant material will be accessed[21]. In addition, the involvement of Tanzanian experts in health economics and critical care will be part of the study team to ensure that no obvious articles or reports are missed.

Due to the complex nature of critical care, a combination of strategies was used to identify key search terms that can capture critical illness and critical care. First, the core concepts that we will search on were identified through the PICO framework: critical care (inpatient services that constitute critical care provision), critical illness, resource use (resource utilisation and cost), setting (Tanzania). Subsequently, to identify the search terms, we reviewed the MESH terms related to the key concepts in Medline, consulted within our expert group and scoped our existing critical care and costing libraries. We also include leading causes of death in Tanzania in the search string (e.g. neonatal disorders, HIV, Malaria and TB[22]), on the premise that these are conditions that have a high risk of critical illness. Variations of the key words will be included in the search string. The concepts and search terms are listed in table 2. Search strategies were tailored to search the three databases.

Table 2: Key concepts and search terms identified for the search.

**Concept**	**Search terms**
Inpatient services that can be considered to form part of critical care provision	***Critical care:***<br><br>Critical care, emergency care, critical emergency care, essential emergency and critical care, intensive care, essential critical care, early goal directed therapy, neonatal, acute care, emergency medicine, trauma, emergency medical services, ICU, Peri-operative care, maternal emergencies.<br><br>***General inpatient care:***<br><br>General ward care, general inpatient care, general care, hospital care<br><br>***Oxygen therapy:***<br><br>Oxygen therapy/provision, artificial ventilation, mechanical ventilation Non-invasive ventilation (NIV), Continuous Positive Airway Pressure (CPAP), High flow (nasal) oxygen (HFO, HFNO)

For peer review only - http://bmjopen.bmj.com/site/about/guidelines.xhtml

Critical illness	***Critical illness:*** Critical illness, sick children, acute paediatrics, emergency obstetric care, poly-trauma, Severe illness, Life-threatening illness, Acute illness, Emergency illness ***Leading causes of mortality in Tanzania:*** HIV, Malaria, TB, sepsis, trauma, burns, pneumonia, emergency surgery, shock, haemorrhage, respiratory failure, coma, unconsciousness, meningitis, choking, anuria, acute kidney injury
Resource use	Cost, expen-, spending, invest- Financ-, financial burden, financial impact, financial consequence, economic, economic burden, economic impact, economic consequence Direct cost, indirect cost, medical cost, non-medical cost, nonmedical cost, opportunity cost Resource, resource use, resource utilisation, resource utilization, health service use, health service utilisation, health service utilization Provider cost, health system cost, hospital cost, system cost, provider cost, hospital cost, societal cost, insurance, reimburse-, cost of illness, cost analysis, economic modelling
Tanzania	Tanzania

Eligibility criteria

A study will be considered eligible if it is published in English and reports on forms/types of critical care, services offered under critical care, resources and or costs incurred by the health system or health providers in providing critical care in Tanzania. A detailed inclusion exclusion criterion can be found in appendix 1.

Study Selection

The PRISMA guidelines[23] will be followed in the selection process. The number of articles retrieved will be listed and uploaded to Rayyan QCRI software which will be used to identify and remove duplicates[24]. Eligibility of identified studies will be assessed independently by researchers LG, HS, SG, PT and JK by first reviewing the title and abstract. All conflicts will be discussed and agreed upon through consensus with third researcher (LG or HS) when applicable. The eligibility of each of the remaining studies will be decided on through full text review of each paper by at least two researchers (SG, JK, HS, PT, LG).

Data extraction process

A data extraction form including author, year of publication, context (location, setting- urban or rural, type of facility, level of facility), critical care services offered, critical care equipment available, costing

For peer review only

perspective, costing year, currency used, type of provider, payer, source of cost data, costing time frame, direct medical costs, resources used, cost ingredients.

Simultaneously during the data extraction process, studies will be assessed for similarity in cost ingredients to be included in the quantitative (cost) synthesis.

Risk of bias in individual studies

The quality of the included articles will be assessed using the Reference Case for Estimating the Costs of Global Health Services and Interventions[25], which provides a framework for quality assessment and data extraction. While standard practice for review of economic evaluations is the Consolidated Health Economic Evaluation Reporting Standards (CHEERS) tool, this does not provide sufficient information to assess the quality of cost studies. Any discrepancies will be addressed by a joint re-evaluation of the article among all authors.

Summary measures and synthesis of results

The summary measures used in this study will be descriptive statistics such as frequencies, mean and median critical care services, costs and quantities identified during the data extraction. Confidence intervals of the point estimates of costs and resource quantities will be captured when reported by the included studies.

Synthesis of the costs and resources will be performed, where costs can be standardised, to estimate average costs/resources per patient of the different components of critical care.

Patient and public involvement

Patients and the public will not be involved in this research, as this study will focus on reviewing secondary publicly accessible data.

Ethics and Dissemination

This study is a review of secondary data, and is part of the Provision Of Essential Treatment In Critical Illness in the context of the COVID-19 pandemic (POETIC). Ethical clearance was sought and granted from The Tanzanian National Institute of Medical Research (reference: NIMR/HQ/R.8a/Vol. IX/3537) and London School of Hygiene and Tropical Medicine (LSHTM ethics ref: 22866).

Following completion of the systematic review, we shall assess whether the research aim and questions have been met and answered respectively using the Grading of Recommendations, Assessment, Development and Evaluations frame work[26].

We aim to publish our findings in a peer reviewed journal as an open access article in addition to presenting the findings at conferences and public scientific gatherings. These findings will also be a basis and provide special input for a cost effectiveness study of EECC in Tanzania.

References

1. Baker T, Schell C O, Brun Petersen D, et al. Essential care of critical illness must not be forgotten in the COVID-19 pandemic. *Lancet*. 2020;395:1253-1254. doi:10.1016/S0140-6736(20)30793-5

For peer review only

2. Barasa E, Kairu A, Nganga W, et al. Examining Unit Costs for COVID-19 Case Management in Kenya. *medRxiv*. Published online October 13, 2020:2020.10.08.20209684. doi:10.1101/2020.10.08.20209684

3. World Bank. World Bank facilitates immediate support and resources for the COVID-19 (Coronavirus) emergency in Bolivia. Published April 2, 2020. Accessed December 16, 2020. https://www.worldbank.org/en/news/press-release/2020/04/02/el-banco-mundial-facilita-apoyo-y-recursos-inmediatos-para-atender-la-emergencia-por-el-covid-19-coronavirus-en-bolivia

4. The World Bank. *The World Bank Annual Report 2020: Supporting Countries in Unprecedented Time*.; 2020. Accessed December 16, 2020. https://www.worldbank.org/en/about/annual-report/covid-response

5. National Institute for Health and Care Excellence (NICE). What “critical care” means | Information for the public | Rehabilitation after critical illness in adults | Guidance | NICE. Published March 25, 2009. Accessed November 1, 2020. https://www.nice.org.uk/guidance/cg83/ifp/chapter/What-critical-care-means

6. Zimmerman JE, Kramer AA, Knaus WA. Changes in hospital mortality for United States intensive care unit admissions from 1988 to 2012. *Crit Care*. 2013;17(2):R81. doi:10.1186/cc12695

7. Murthy S, Adhikari NK. ATS REPORTS Global Health Care of the Critically Ill in Low-Resource Settings. *Ann Am Thorac Soc*. 2013;10(5):509-513. doi:10.1513/AnnalsATS.201307-246OT

8. Vukoja M, Riviello ED, Schultz MJ. Critical care outcomes in resource-limited settings. *Curr Opin Crit Care*. 2018;24(5):421-427. doi:10.1097/MCC.0000000000000528

9. Schell CO, Gerdin Wärnberg M, Hvarfner A, et al. The global need for essential emergency and critical care. *Crit Care*. 2018;22(1):1-5. doi:10.1186/s13054-018-2219-2

10. Turner HC, Van Hao N, Yacoub S, et al. Achieving affordable critical care in low-income and middle-income countries Commentary Handling editor Seye Abimbola. *BMJ Glob Heal*. 2019;4:1675. doi:10.1136/bmjgh-2019-001675

11. Adhikari NKJ, Fowler RA, Bhagwanjee S, Rubenfeld GD. Critical care and the global burden of critical illness in adults. *Lancet*. 2010;376(9749):1339-1346. doi:10.1016/S0140-6736(10)60446-1

12. Ma X, Vervoort D. Critical care capacity during the COVID-19 pandemic: Global availability of intensive care beds. *J Crit Care*. 2020;58(January):96-97. http://europepmc.org/backend/ptpmcrender.fcgi?accid=PMC7194590&blobtype=pdf

13. Dart PJ, Kinnear J, Bould MD, Mwansa SL, Rakhda Z, Snell D. An evaluation of inpatient morbidity and critical care provision in Zambia. *Anaesthesia*. 2017;72(2):172-180. doi:10.1111/anae.13709

14. Kayambankadzanja RK, Schell CO, Namboya F, et al. The prevalence and outcomes of sepsis in adult patients in two hospitals in Malawi. *Am J Trop Med Hyg*. 2020;102(4):896-901. doi:10.4269/ajtmh.19-0320

15. Lone NI, Seretny M, Wild SH, Rowan KM, Murray GD, Walsh TS. Surviving intensive care: A systematic review of healthcare resource use after hospital discharge. *Crit Care Med*. 2013;41(8):1832-1843. doi:10.1097/CCM.0b013e31828a409c

16. Abu Al-Saad N, Skedgel C, Nortje J. Principles of resource allocation in critical care. *BJA Educ*.

For peer review only

2017;17(12):390-395. doi:10.1093/bjaed/mkx029

17. Woo BFY, Lee JXY, Tam WWS. The impact of the advanced practice nursing role on quality of care, clinical outcomes, patient satisfaction, and cost in the emergency and critical care settings: A systematic review. *Hum Resour Health*. 2017;15(1):1-22. doi:10.1186/s12960-017-0237-9

18. Negrini D, Sheppard L, Mills GH, et al. International Programme for Resource Use in Critical Care (IPOC) - A methodology and initial results of cost and provision in four European countries. *Acta Anaesthesiol Scand*. 2006;50(1):72-79. doi:10.1111/j.1399-6576.2006.00901.x

19. Shamseer L, Moher D, Clarke M, et al. Preferred reporting items for systematic review and meta-analysis protocols (prisma-p) 2015: Elaboration and explanation. *BMJ*. 2015;349(January):1-25. doi:10.1136/bmj.g7647

20. Higgins J, Thomas J, Chandler J, et al. *Cochrane Handbook for Systematic Reviews of Interventions*. Cochrane; 2020. https://training.cochrane.org/handbook/current

21. Savilaakso S, Garcia C, Garcia-Ulloa J, et al. Systematic review of effects on biodiversity from oil palm production. *Environ Evid*. 2014;3(1):4. doi:10.1186/2047-2382-3-4

22. Abbafati C, Machado DB, Cislaghi B, et al. Global burden of 369 diseases and injuries in 204 countries and territories, 1990–2019: a systematic analysis for the Global Burden of Disease Study 2019. *Lancet*. 2020;396(10258):1204-1222. doi:10.1016/S0140-6736(20)30925-9

23. Moher D, Liberati A, Tetzlaff J, et al. Preferred reporting items for systematic reviews and meta-analyses: The PRISMA statement (Chinese edition). *J Chinese Integr Med*. 2009;7(9):889-896. doi:10.3736/jcim20090918

24. Ouzzani M, Hammady H, Fedorowicz Z, Elmagarmid A. Rayyan-a web and mobile app for systematic reviews. *Syst Rev*. 2016;5(1). doi:10.1186/s13643-016-0384-4

25. Vassall A, Sweeney S, Kahn JG, et al. *Reference Case for Estimating the Costs of Global Health Services and Interventions*.

26. Guyatt G, Oxman AD, Akl EA, et al. GRADE guidelines: 1. Introduction - GRADE evidence profiles and summary of findings tables. *J Clin Epidemiol*. 2011;64(4):383-394. doi:10.1016/j.jclinepi.2010.04.026

For peer review only

Authors' contributions

JK, LG, HS conceptualized the literature review. The first author developed the protocol and LG, HS, SG, PT, TB, COS, KK and AJ reviewed the protocol.

Funding statement

This work was supported by the Wellcome Trust [221571/Z/20/Z], as part of the 'Innovation in low-and middle-income countries' Flagship.

Competing interests statement

Authors declare no competing interests and the funders did not in any way influence the development of the development of this protocol.

APPENDIX 1

Inclusion and exclusion criteria

Inclusion criterion

For a study to be included it should fulfil the following criterion

- Published in English in a peer reviewed journal
- Reports forms or types of critical care offered, critical care services offered and or costs and resources used in the provision of care in Tanzania
- Includes costs from a provider perspective
- Articles published from 2010

Exclusion criterion

Studies will be excluded in case of the following;

- Published in any other languages except English
- Studies carried out outside Tanzania
- Studies with costs estimated from patient perspective
- Studies without full text versions
- Studies published before 2010

For peer review only

BMJ Open

Resource availability, utilization and cost in the provision of critical care in Tanzania: A protocol for a systematic review

Journal:	*BMJ Open*
Manuscript ID	bmjopen-2021-050881.R1
Article Type:	Protocol
Date Submitted by the Author:	17-Jun-2021
Complete List of Authors:	Kazibwe, Joseph; Imperial College London, Department of Infectious Disease Epidemiology<br>Shah, Hiral A.; Imperial College London, Department of Infectious Disease Epidemiology; Center for Global Development, Department of Infectious Disease Epidemiology<br>Kuwawenaruwa, A; Ifakara Health Institute<br>Schell, Carl Otto; Karolinska Institutet, Department of Global Public Health; Uppsala University, Department of Global Public Health<br>Khalid, Karima; Ifakara Health Institute; Muhimbili University of Health and Allied Sciences<br>Tran, Phuong Bich; University of Antwerp, Department of Family and Population Health<br>Ghosh, Srobana; Center for Global Development<br>Baker, Tim; Karolinska Institutet, Department of Global Public Health<br>GUINNESS, LORNA; Center for Global Development; London School of Hygiene and Tropical Medicine, GLOBAL HEALTH & DEVELOPMENT
**Primary Subject Heading**:	Emergency medicine
Secondary Subject Heading:	Cardiovascular medicine, Communication
Keywords:	INTENSIVE & CRITICAL CARE, HEALTH ECONOMICS, Health policy < HEALTH SERVICES ADMINISTRATION & MANAGEMENT

SCHOLARONE™ Manuscripts

I, the Submitting Author has the right to grant and does grant on behalf of all authors of the Work (as defined in the below author licence), an exclusive licence and/or a non-exclusive licence for contributions from authors who are: i) UK Crown employees; ii) where BMJ has agreed a CC-BY licence shall apply, and/or iii) in accordance with the terms applicable for US Federal Government officers or employees acting as part of their official duties; on a worldwide, perpetual, irrevocable, royalty-free basis to BMJ Publishing Group Ltd ("BMJ") its licensees and where the relevant Journal is co-owned by BMJ to the co-owners of the Journal, to publish the Work in this journal and any other BMJ products and to exploit all rights, as set out in our licence.

The Submitting Author accepts and understands that any supply made under these terms is made by BMJ to the Submitting Author unless you are acting as an employee on behalf of your employer or a postgraduate student of an affiliated institution which is paying any applicable article publishing charge ("APC") for Open Access articles. Where the Submitting Author wishes to make the Work available on an Open Access basis (and intends to pay the relevant APC), the terms of reuse of such Open Access shall be governed by a Creative Commons licence – details of these licences and which Creative Commons licence will apply to this Work are set out in our licence referred to above.

Other than as permitted in any relevant BMJ Author's Self Archiving Policies, I confirm this Work has not been accepted for publication elsewhere, is not being considered for publication elsewhere and does not duplicate material already published. I confirm all authors consent to publication of this Work and authorise the granting of this licence.

For peer review only

Resource availability, utilization and cost in the provision of critical care in Tanzania: A protocol for a systematic review

For peer review only

Corresponding Author

Joseph Kazibwe
Department of Infectious Disease Epidemiology
Imperial College London, London
Norfolk Place, London W2 1PG – UK
j.kazibwe@imperial.ac.uk
+447561876477

Authors and affiliation

Joseph Kazibwe[1], Hiral A. Shah[1,5], August Kuwawenaruwa[2], Carl Otto Schell[7,8], Karima Khalid[2,3], Phuong Bich Tran[4], Srobana Ghosh[5], Tim Baker[2,6], Lorna Guinness[5]

1. Department of Infectious Disease Epidemiology, Imperial College London, London – UK
2. Ifakara Health Institute, Dar es Salaam – Tanzania
3. Muhimbili University of Health and Allied Sciences, Dar es Salaam – Tanzania
4. Department of Family and Population Health (FAMPOP), University of Antwerp, Antwerp - Belgium
5. Center for Global Development (CGD), London – UK
6. Department of Clinical Research, London School of Hygiene and Tropical Medicine, UK
7. Department of Global Public Health, Karolinska Institutet, Stockholm, Sweden
8. Centre for Clinical Research Sörmland, Uppsala University, Eskilstuna, Sweden

Word count (excluding title page, abstract, references, figures and tables): 1,939

Key words: Critical care, costing, utilization of resources in critical care, forms of critical care, critical illness, low resource settings

Abstract

Introduction

Critical care is essential in saving lives of those that are critically ill, however, provision of critical care can be costly and heterogenous across lower resource settings. This paper describes the protocol for a systematic review of the literature that aims to identify the reported costs and resources available for the provision of critical care and the forms of critical care provision in Tanzania.

Methods and analysis

The review will follow the Preferred Reporting Items for Systematic Reviews and Meta-Analyses (PRISMA) guidelines. Three databases (MEDLINE, Embase and Global Health) will be searched to identify articles that report the forms of critical care, resources used in the provision of critical care in Tanzania, their availability and the associated costs. The search strategy will be developed from four key concepts; critical care provision, critical illness, resource use, Tanzania. The articles that fulfil the inclusion and exclusion criteria will be assessed for quality using the Reference Case for Estimating the Costs of Global Health Services and Interventions checklist. The extracted data will be summarized using descriptive statistics including frequencies, mean and median of the quantity and costs of resources used in the components of critical care services, depending on the data availability.

Ethics and dissemination

This study is a review of secondary data and ethical clearance was sought from and granted by the Tanzanian National Institute of Medical Research (reference: NIMR/HQ/R.8a/Vol. IX/3537) and London School of Hygiene and Tropical Medicine (LSHTM ethics ref: 22866). We will publish the review in a peer reviewed journal as an open access article in addition to presenting the findings at conferences and public scientific gatherings.

Strengths and weaknesses of the proposed study

- This is the first systematic review of the literature around the costs and resources used in critical care and forms of critical care in a lower resource setting such as Tanzania. This study will provide an overview on the current evidence base on resources and costs for decision-making in critical care in Tanzania and insight for other low resource settings.
- The search strategy broadly and comprehensively includes studies on critical care and costs of inpatient care in Tanzania increasing chances of including all published studies on the subject.
- This study has a fully developed population, intervention, comparison and outcome (PICO) framework.
- The study will include only articles published in English.
- There is a chance that there may be limited relevant data on Tanzania in that there may also be a publication bias where published literature focuses on specific diseases (e.g. malaria, pneumonia).

Definitions

Forms of critical care are the different levels of critical care services (according to level of advancement) that can be offered to a critically ill patient that can range from basic services like oxygen therapy in general wards to mechanical ventilation in ICUs.

For peer review only

For peer review only - http://bmjopen.bmj.com/site/about/guidelines.xhtml

Resources (in this study) are the physical items, material or equipment used in the provision of critical care in a given setting. Resources will be classified using a standard of classification, that is; by input (human resources, consumables etc) or by activities (diagnostics, bed days etc) depending on the data available.

Background

The COVID19 pandemic is now synonymous with being a critical care crisis with both high income and low-and-middle income countries (LMICs) struggling to meet demand for critical care services.[1,2] This has led to the care of critical illness becoming an urgent issue and a point of focus in global health with stakeholders providing recommendations on what interventions should be scaled up, while knowledge on the potential costs involved remains sparse[1,3,4].

Critical care entails the care given to a patient in need of specialist monitoring, treatment and attention, for example for a patient with a life-threatening illness or injury[5]. Over the years, there have been great advances in the provision of critical care for example invasive and non-invasive monitoring techniques, mechanical ventilation (MV), and renal replacement therapy, among others which have resulted in reduced mortality rates over time among patients with critical illness especially in high income countries[6].

Despite the advances made in the provision of critical care in LMICs[7–9], the limited availability of intensive care units (ICUs) and the low numbers of trained personnel in such settings persist. In addition, the frequent power cuts and inadequate supply of oxygen in some areas make advanced care challenging. The equipment in an ICU can be expensive and may not be affordable or available in many LMICs with an already constrained healthcare budget[10,11]. In a global mapping of ICU bed capacity, the ICU bed density in the majority of Sub-Saharan African countries was reported to be below 1.0 ICU bed per 100,000 population as compared with more than 25 in Germany and the USA[12]. Even where ICUs are available in LMICs settings, the services offered are expensive, making them inaccessible and unaffordable for the majority of critically ill patients. This limited availability of critical care services in LMICs has been linked to the high ICU related deaths of between 30%-80% of all ICU admissions[8]. In fact, most critically ill patients are cared for in general hospital wards[13,14].

Given the constraints that LMICs face when having to provide critical care, recent research has proposed the concept of Essential Emergency and Critical Care (EECC) which focuses on providing low-cost care for critical illness in any place within the hospital[1,9]. EECC consists of effective life-saving actions of low cost and complexity, is appropriate for all hospital settings and for all critically ill patients irrespective of their age, sex, location or underlying medical condition[9]. EECC has been proposed as a key component in universal health coverage (UHC). Research is ongoing into the policy and economic aspects of EECC in Tanzania and Kenya to inform EECC implementation in these and other low resourced health system settings.

To inform this work, it is necessary to review the existing knowledge about the current resource availability, utilization and cost in the provision of critical care in Tanzania. Several studies have been conducted to estimate the resource use and cost of critical care globally but the majority of these estimates are confined to high income countries and may not be generalizable to the LMICs settings [15–17]. Even studies that look at the resource use and cost of critical care, whose findings are reported as

For peer review only - http://bmjopen.bmj.com/site/about/guidelines.xhtml

international estimates, focus on high income countries for example Germany, Hungary, UK and France[18]. In addition, the majority of studies focus on the costs incurred in an ICU only[17] which may not reflect the resources used for all critically illness, especially in LMICs. Here, we describe the protocol of a systematic review that will identify the existing evidence on the quantification of current resources available and the economic costs in the provision of critical care in Tanzania.

Aim

This paper describes the protocol for a systematic review of the literature that aims to identify the reported costs and resources available for the provision of critical care and the forms of critical care provision in Tanzania.

Research questions

1. What are forms of critical care provided in the health system in Tanzania?
2. What resources are reported to be available and have been utilised in the provision of critical care in Tanzania?
3. What are the reported costs of available resources (including oxygen supplies) used in critical care provision in Tanzania?

Methods

The systematic review will follow the Preferred Reporting Items for Systematic Reviews and Meta-Analyses (PRISMA) guidelines[19] and the protocol was registered with PROSPERO; registration number: CRD42020221923.

The review will use a PICO (Population, Intervention, Comparator, Outcome) framework to enable a comprehensive and unbiased search of existing literature and identify the key search terms[20] (see table 1) . Critical care as a concept does not have an internationally accepted definition. The review defines critical care as the components of care that support a critically ill patient's vital organ functions where critical illness is a state of ill health with vital organ dysfunction, a high risk of imminent death if action is not taken and the potential for reversibility. A critically ill patient is one with acute need for life saving organ support. Critical care is ideally team-based and multi-professional and can be given in any location. Critical care can be high-intensity e.g mechanical ventilation, requiring highly specialised services, or it can be low intensity resuscitative and supportive care e.g oxygen provided using a face mask.

Table 1: PICO (Population, Intervention, Comparator, Outcome) Framework used to inform the search strategy.

Element	Description
Population	Any patient in need of critical care
Intervention	Inpatient services that could form part of critical care provision
Comparison	No critical care

For peer review only

Outcomes	Critical care services available<br><br>Cost of the different inputs to critical care provision per patient<br><br>Quantity and type of resources used in provision of critical care per patient

Search strategy

Electronic databases (Medline (Ovid), Embase (embase.com) and Global Health) will be systematically searched for articles published between 2010 to present. A ten-year time frame will be considered to ensure that findings reflect the present-day resource utilization paradigm in critical care provision as resources used and their costs vary with the nature of technology and clinical guidelines in place. Bibliographies of included articles will be reviewed to find relevant articles that fulfil the inclusion criteria but not yet identified. Authors whose abstracts will be considered for inclusion, but the full text is not accessible online, will be contacted and requested for article access. Google will also be used to search for published articles that may not have been indexed within the databases. As internet search engines typically return several thousand results, the searches will be restricted to the first fifty hits and links to potentially relevant material will be accessed[21]. The search will be done in anonymous mode to ensure that we do not pick up searcher's embedded preferences. In addition, the involvement of Tanzanian experts in health economics and critical care will be part of the study team to ensure that no obvious articles or reports are missed.

Due to the complex nature of critical care, a combination of strategies was used to identify key search terms that can capture critical illness and critical care. First, the core concepts that we will search on were identified through the PICO framework: critical care (inpatient services that constitute critical care provision), critical illness, resource use (resource utilisation and cost), setting (Tanzania). Subsequently, to identify the search terms, we reviewed the MESH terms related to the key concepts in Medline, consulted within our expert group and scoped our existing critical care and costing libraries. We also include leading causes of death in Tanzania in the search string (e.g. neonatal disorders, HIV, Malaria and tuberculosis (TB)[22]), on the premise that these are conditions that have a high risk of critical illness. Variations of the key words will be included in the search string. The concepts and search terms are listed in table 2. Search strategies were tailored to search the three databases. Search strategies for each database are available in appendix 1.

Table 2: Key concepts and search terms identified for the search.

**Concept**	**Search terms**
Inpatient services that can be considered to form part of critical care provision	***Critical care:***<br><br>Critical care, emergency care, critical emergency care, essential emergency and critical care, intensive care, essential critical care, early goal directed therapy, neonatal, acute care, emergency medicine, trauma, emergency medical services, ICU, Peri-operative care, maternal emergencies, cardiovascular/inotropic support, renal support

For peer review only - http://bmjopen.bmj.com/site/about/guidelines.xhtml

	General inpatient care: General ward care, general inpatient care, general care, hospital care ***Oxygen therapy:*** Oxygen therapy/provision, artificial ventilation, mechanical ventilation Non-invasive ventilation (NIV), Continuous Positive Airway Pressure (CPAP), High flow (nasal) oxygen (HFO, HFNO)
Critical illness	***Critical illness:*** Critical illness, sick children, acute paediatrics, emergency obstetric care, poly-trauma, Severe illness, Life-threatening illness, Acute illness, Emergency illness ***Leading causes of mortality in Tanzania:*** HIV, Malaria, TB, sepsis, trauma, burns, pneumonia, emergency surgery, shock, haemorrhage, respiratory failure, coma, unconsciousness, meningitis, choking, anuria, acute kidney injury
Resource use	Cost, expen-, spending, invest- Financ-, financial burden, financial impact, financial consequence, economic, economic burden, economic impact, economic consequence Direct cost, indirect cost, medical cost, non-medical cost, nonmedical cost, opportunity cost Resource, resource use, resource utilisation, resource utilization, health service use, health service utilisation, health service utilization Provider cost, health system cost, hospital cost, system cost, provider cost, hospital cost, societal cost, insurance, reimburse-, cost of illness, cost analysis, economic modelling
Tanzania	Tanzania

Eligibility criteria

A study will be considered eligible if it is published in English and reports on forms/types of critical care, services offered under critical care, resources and or costs incurred by the health system or health providers in providing critical care in Tanzania. A detailed inclusion exclusion criterion can be found in appendix 2.

Study Selection

For peer review only - http://bmjopen.bmj.com/site/about/guidelines.xhtml

The PRISMA guidelines[23] will be followed in the selection process. The number of articles retrieved will be listed and uploaded to Rayyan QCRI software which will be used to identify and remove duplicates[24]. Eligibility of identified studies will be assessed independently by researchers LG, HS, SG, PT and JK by first reviewing the title and abstract. All conflicts will be discussed and agreed upon through consensus with third researcher (LG or HS) when applicable. The eligibility of each of the remaining studies will be decided on through full text review of each paper by at least two researchers (SG, JK, HS, PT, LG).

Data extraction process

A data extraction form including author, year of publication, context (location, setting- urban or rural, type of facility, level of facility), critical care services offered (including special critical care services), critical care equipment available, costing perspective, costing year, currency used, type of provider, payer, source of cost data, costing time frame, direct medical costs, resources used, cost ingredients will be developed.

Simultaneously during the data extraction process, studies will be assessed for similarity in cost ingredients to be included in the quantitative (cost) synthesis.

Risk of bias in individual studies

The quality of the included articles will be assessed using the Reference Case for Estimating the Costs of Global Health Services and Interventions[25], which provides a framework for quality assessment and data extraction. While standard practice for review of economic evaluations is the Consolidated Health Economic Evaluation Reporting Standards (CHEERS) tool, this does not provide sufficient information to assess the quality of cost studies. Any discrepancies will be addressed by a joint re-evaluation of the article among all authors. The appraisal checklist[26] will be used to assess the similarity between the reported costs. For studies that do not report costs, the Newcastle Ottawa Scale will be used to assess their quality[27].

Summary measures and synthesis of results

Studies will be grouped into two; 1) those that report on costs and 2) those that report on forms of critical care. The summary measures used in this study will be descriptive statistics such as frequencies, mean and median critical care services, costs and quantities identified during the data extraction. Confidence intervals of the point estimates of costs and resource quantities will be captured when reported by the included studies. Group analysis of the results will be done along level of health facility/hospital, health services offered, cost reported, data sources, study designs, geographical location (rural and urban).

Synthesis of the costs and resources will be performed. Costs, average costs/resources per patient of the different components of critical care will be estimated and converted to 2019 USD and TZS, using the World Bank GDP deflators[28]. The costs will be presented per the different components of critical care. A synthesis without meta-analysis (SWiM) checklist[29] will be used to guide the synthesis. A detailed checklist is available as an appendix (appendix 3).

Timeline

The study is expected to be completed by 31st October 2021.

Patient and public involvement

Patients and the public will not be involved in this research, as this study will focus on reviewing secondary publicly accessible data.

Ethics and Dissemination

For peer review only - http://bmjopen.bmj.com/site/about/guidelines.xhtml

This study is a review of secondary data, and ethical clearance was sought from and granted by The Tanzanian National Institute of Medical Research (reference: NIMR/HQ/R.8a/Vol. IX/3537) and London School of Hygiene and Tropical Medicine (LSHTM ethics ref: 22866).

Following completion of the systematic review, we shall assess whether the research aim and questions have been met and answered respectively using the Grading of Recommendations, Assessment, Development and Evaluations frame work[30].

We aim to publish our findings in a peer reviewed journal as an open access article in addition to presenting the findings at conferences and public scientific gatherings. These findings will also be a basis and provide special input for a cost effectiveness study of EECC in Tanzania.

For peer review only

References

1. Baker T, Otto Schell C, Brun Petersen D, et al. Essential care of critical illness must not be forgotten in the COVID-19 pandemic. *Lancet*. 2020;395:1253-1254. doi:10.1016/S0140-6736(20)30793-5
2. Barasa E, Kairu A, Nganga W, et al. Examining Unit Costs for COVID-19 Case Management in Kenya. *medRxiv*. Published online October 13, 2020:2020.10.08.20209684. doi:10.1101/2020.10.08.20209684
3. World Bank. World Bank facilitates immediate support and resources for the COVID-19 (Coronavirus) emergency in Bolivia. Published April 2, 2020. Accessed December 16, 2020. https://www.worldbank.org/en/news/press-release/2020/04/02/el-banco-mundial-facilita-apoyo-y-recursos-inmediatos-para-atender-la-emergencia-por-el-covid-19-coronavirus-en-bolivia
4. The World Bank. *The World Bank Annual Report 2020: Supporting Countries in Unprecedented Time*.; 2020. Accessed December 16, 2020. https://www.worldbank.org/en/about/annual-report/covid-response
5. National Institute for Health and Care Excellence (NICE). What "critical care" means | Information for the public | Rehabilitation after critical illness in adults | Guidance | NICE. Published March 25, 2009. Accessed November 1, 2020. https://www.nice.org.uk/guidance/cg83/ifp/chapter/What-critical-care-means
6. Zimmerman JE, Kramer AA, Knaus WA. Changes in hospital mortality for United States intensive care unit admissions from 1988 to 2012. *Crit Care*. 2013;17(2):R81. doi:10.1186/cc12695
7. Murthy S, Adhikari NK. ATS REPORTS Global Health Care of the Critically Ill in Low-Resource Settings. *Ann Am Thorac Soc*. 2013;10(5):509-513. doi:10.1513/AnnalsATS.201307-246OT
8. Vukoja M, Riviello ED, Schultz MJ. Critical care outcomes in resource-limited settings. *Curr Opin Crit Care*. 2018;24(5):421-427. doi:10.1097/MCC.0000000000000528
9. Schell CO, Gerdin Wärnberg M, Hvarfner A, et al. The global need for essential emergency and critical care. *Crit Care*. 2018;22(1):1-5. doi:10.1186/s13054-018-2219-2
10. Turner HC, Van Hao N, Yacoub S, et al. Achieving affordable critical care in low-income and middle-income countries Commentary Handling editor Seye Abimbola. *BMJ Glob Heal*. 2019;4:1675. doi:10.1136/bmjgh-2019-001675

For peer review only - http://bmjopen.bmj.com/site/about/guidelines.xhtml

11. Adhikari NKJ, Fowler RA, Bhagwanjee S, Rubenfeld GD. Critical care and the global burden of critical illness in adults. *Lancet*. 2010;376(9749):1339-1346. doi:10.1016/S0140-6736(10)60446-1

12. Ma X, Vervoort D. Critical care capacity during the COVID-19 pandemic: Global availability of intensive care beds. *J Crit Care*. 2020;58(January):96-97. http://europepmc.org/backend/ptpmcrender.fcgi?accid=PMC7194590&blobtype=pdf

13. Dart PJ, Kinnear J, Bould MD, Mwansa SL, Rakhda Z, Snell D. An evaluation of inpatient morbidity and critical care provision in Zambia. *Anaesthesia*. 2017;72(2):172-180. doi:10.1111/anae.13709

14. Kayambankadzanja RK, Schell CO, Namboya F, et al. The prevalence and outcomes of sepsis in adult patients in two hospitals in Malawi. *Am J Trop Med Hyg*. 2020;102(4):896-901. doi:10.4269/ajtmh.19-0320

15. Lone NI, Seretny M, Wild SH, Rowan KM, Murray GD, Walsh TS. Surviving intensive care: A systematic review of healthcare resource use after hospital discharge. *Crit Care Med*. 2013;41(8):1832-1843. doi:10.1097/CCM.0b013e31828a409c

16. Abu Al-Saad N, Skedgel C, Nortje J. Principles of resource allocation in critical care. *BJA Educ*. 2017;17(12):390-395. doi:10.1093/bjaed/mkx029

17. Woo BFY, Lee JXY, Tam WWS. The impact of the advanced practice nursing role on quality of care, clinical outcomes, patient satisfaction, and cost in the emergency and critical care settings: A systematic review. *Hum Resour Health*. 2017;15(1):1-22. doi:10.1186/s12960-017-0237-9

18. Negrini D, Sheppard L, Mills GH, et al. International Programme for Resource Use in Critical Care (IPOC) - A methodology and initial results of cost and provision in four European countries. *Acta Anaesthesiol Scand*. 2006;50(1):72-79. doi:10.1111/j.1399-6576.2006.00901.x

19. Shamseer L, Moher D, Clarke M, et al. Preferred reporting items for systematic review and meta-analysis protocols (prisma-p) 2015: Elaboration and explanation. *BMJ*. 2015;349(January):1-25. doi:10.1136/bmj.g7647

20. Higgins J, Thomas J, Chandler J, et al. *Cochrane Handbook for Systematic Reviews of Interventions*. Cochrane; 2020. https://training.cochrane.org/handbook/current

21. Savilaakso S, Garcia C, Garcia-Ulloa J, et al. Systematic review of effects on biodiversity from oil palm production. *Environ Evid*. 2014;3(1):4. doi:10.1186/2047-2382-3-4

22. Abbafati C, Machado DB, Cislaghi B, et al. Global burden of 369 diseases and injuries in 204 countries and territories, 1990–2019: a systematic analysis for the Global Burden of Disease Study 2019. *Lancet*. 2020;396(10258):1204-1222. doi:10.1016/S0140-6736(20)30925-9

23. Moher D, Liberati A, Tetzlaff J, et al. Preferred reporting items for systematic reviews and meta-analyses: The PRISMA statement (Chinese edition). *J Chinese Integr Med*. 2009;7(9):889-896. doi:10.3736/jcim20090918

24. Ouzzani M, Hammady H, Fedorowicz Z, Elmagarmid A. Rayyan-a web and mobile app for systematic reviews. *Syst Rev*. 2016;5(1). doi:10.1186/s13643-016-0384-4

25. Vassall A, Sweeney S, Kahn JG, et al. *Reference Case for Estimating the Costs of Global Health Services and Interventions*.

26. Adawiyah R al, Saweri OPM, Boettiger DC, et al. The costs of scaling up HIV and syphilis testing in

For peer review only

low- and middle-income countries: a systematic review. *Health Policy Plan*. 2021;2021:1-16. doi:10.1093/heapol/czab030

27. Wells G, Shea B, O'Connell D, et al. The Newcastle-Ottawa Scale (NOS) for assessing the quality of nonrandomised studies in meta-analyses. Ottawa Hospital Research Institute . Published 2019. Accessed February 23, 2021. http://www.ohri.ca/programs/clinical_epidemiology/oxford.asp

28. World bank. GDP deflator (base year varies by country) - Tanzania . Accessed June 2, 2021. https://data.worldbank.org/indicator/NY.GDP.DEFL.ZS?locations=TZ

29. Campbell M, McKenzie JE, Sowden A, et al. Synthesis without meta-analysis (SWiM) in systematic reviews: Reporting guideline. *BMJ*. 2020;368. doi:10.1136/bmj.l6890

30. Guyatt G, Oxman AD, Akl EA, et al. GRADE guidelines: 1. Introduction - GRADE evidence profiles and summary of findings tables. *J Clin Epidemiol*. 2011;64(4):383-394. doi:10.1016/j.jclinepi.2010.04.026

Authors' contributions

All authors made sufficient contribution to the development of manuscript in line with ICMJE criteria. Below is the detailed breakdown of their contribution.

Concept and design: JK, HS, LG, PT

Development of first draft: JK

Acquisition of data: N/A

Analysis and interpretation of data: N/A

Drafting of the manuscript: JK, LG, HS, SG, PT, COS, KK and AK

Critical revision of paper for important intellectual content: LG, HS, SG, PT, TB, COS, KK and AK

Statistical analysis: N/A

Provision of study materials: JK, TB, LG, PT, COS, KK and AK

Obtaining funding: TB, COS

Administrative and technical support: JK, LG, HS, SG, PT, TB, COS, KK and AK

Supervision: LG, HS

Funding statement

This work was supported by the Wellcome Trust [221571/Z/20/Z], as part of the 'Innovation in low-and middle-income countries' Flagship.

Competing interests statement

Authors declare no competing interests and the funders did not in any way influence the development of the development of this protocol.

For peer review only

For peer review only - http://bmjopen.bmj.com/site/about/guidelines.xhtml

Appendix 1: Search Strategies

1) Medline search strategy

	Interface: Ovid MEDLINE<br>Date of Search:<br>Number of hits:
**Number**	**Search**
1	(((Critical care or emergency care or critical emergency care or essential emergency) and critical care) or intensive care or early goal directed therapy or neonatal or acute care or emergency medicine or trauma or emergency medical services or ICU or Peri-operative care or maternal emergencies or cardiovascular support or inotropic support or renal support).mp.
2	(General ward care or general inpatient care or hospital care or Oxygen therapy or artificial ventilation or mechanical ventilation or Non-invasive ventilation or Continuous Positive Airway Pressure or High flow nasal oxygen or HFO or HFNO).mp.
3	(Critical illness or sick children or acute paediatrics or emergency obstetric care or poly-trauma or Severe illness or Life-threatening illness or Acute illness or HIV or Malaria or TB or sepsis or trauma or burns or pneumonia or emergency surgery or shock or haemorrhage or respiratory failure or coma or unconsciousness or meningitis or choking or anuria or acute kidney injury).mp.
4	(Cost or expen or spending or invest- or Financ or financial burden or financial impact or financial consequence or economic or economic burden or economic impact or economic consequence or Direct cost or indirect cost or medical cost or non-medical cost or nonmedical cost or opportunity cost or Resource or resource or resource utilisation or resource utilization or health service utilisation or health service utilization or Provider cost or health system cost or hospital cost or system cost or provider cost or hospital cost or societal cost or insurance or reimburse- or cost of illness or cost analysis or economic modelling).mp.
5	Tanzania.mp. or exp Tanzania/
6	1 or 2 or 3 or 4
7	5 and 6
8	limit 7 to yr="2010 -Current"

2) Embase search strategy

	Interface: Ovid Embase<br>Date of Search:

For peer review only

	Number of hits:
**Number**	**Search**
1	(((Critical care or emergency care or critical emergency care or essential emergency) and critical care) or intensive care or early goal directed therapy or neonatal or acute care or emergency medicine or trauma or emergency medical services or ICU or Peri-operative care or maternal emergencies or cardiovascular support or inotropic support or renal support).mp.
2	(General ward care or general inpatient care or hospital care or Oxygen therapy or artificial ventilation or mechanical ventilation or Non-invasive ventilation or Continuous Positive Airway Pressure or High flow nasal oxygen or HFO or HFNO).mp.
3	(Critical illness or sick children or acute paediatrics or emergency obstetric care or poly-trauma or Severe illness or Life-threatening illness or Acute illness or HIV or Malaria or TB or sepsis or trauma or burns or pneumonia or emergency surgery or shock or haemorrhage or respiratory failure or coma or unconsciousness or meningitis or choking or anuria or acute kidney injury).mp.
4	(Cost or expen or spending or invest- or Financ or financial burden or financial impact or financial consequence or economic or economic burden or economic impact or economic consequence or Direct cost or indirect cost or medical cost or non-medical cost or nonmedical cost or opportunity cost or Resource or resource or resource utilisation or resource utilization or health service utilisation or health service utilization or Provider cost or health system cost or hospital cost or system cost or provider cost or hospital cost or societal cost or insurance or reimburse- or cost of illness or cost analysis or economic modelling).mp.
5	Tanzania.mp. or exp Tanzania/
6	1 or 2 or 3 or 4
7	5 and 6
8	limit 7 to yr="2010 -Current"

3) Global health Search Strategy

	Interface: Ovid Global Health<br>Date of Search:<br>Number of hits:
**Number**	**Search**
1	(((Critical care or emergency care or critical emergency care or essential emergency) and critical care) or intensive care or early goal directed therapy or neonatal or acute care or emergency medicine or trauma or emergency medical services or ICU or Peri-operative care or maternal emergencies or cardiovascular support or inotropic support or renal support).mp.

For peer review only

2	(General ward care or general inpatient care or hospital care or Oxygen therapy or artificial ventilation or mechanical ventilation or Non-invasive ventilation or Continuous Positive Airway Pressure or High flow nasal oxygen or HFO or HFNO).mp.
3	(Critical illness or sick children or acute paediatrics or emergency obstetric care or poly-trauma or Severe illness or Life-threatening illness or Acute illness or HIV or Malaria or TB or sepsis or trauma or burns or pneumonia or emergency surgery or shock or haemorrhage or respiratory failure or coma or unconsciousness or meningitis or choking or anuria or acute kidney injury).mp.
4	(Cost or expen or spending or invest- or Financ or financial burden or financial impact or financial consequence or economic or economic burden or economic impact or economic consequence or Direct cost or indirect cost or medical cost or non-medical cost or nonmedical cost or opportunity cost or Resource or resource or resource utilisation or resource utilization or health service utilisation or health service utilization or Provider cost or health system cost or hospital cost or system cost or provider cost or hospital cost or societal cost or insurance or reimburse- or cost of illness or cost analysis or economic modelling).mp.
5	Tanzania.mp. or exp Tanzania/
6	1 or 2 or 3 or 4
7	5 and 6
8	limit 7 to yr="2010 -Current"

For peer review only

Appendix 2: Inclusion and exclusion criteria

Inclusion criterion

For a study to be included it should fulfil the following criterion

- Published in English in a peer reviewed journal
- Reports forms or types of critical care offered, critical care services offered and or costs and resources used in the provision of care in Tanzania
- Includes costs from a provider perspective
- Articles published from 2010

Exclusion criterion

Studies will be excluded in case of the following;

- Published in any other languages except English
- Studies carried out outside Tanzania
- Studies with costs estimated from patient perspective
- Studies without full text versions
- Studies published before 2010

For peer review only

Synthesis Without Meta-analysis (SWiM) reporting items

SWiM is intended to complement and be used as an extension to PRISMA			
**SWiM reporting item**	**Item description**	**Page in manuscript where item is reported**	**Other***
Methods			
**1** Grouping studies for synthesis	The studies will be grouped into two; studies that report cost and studies that report on forms of critical care. For the studies that report costs of critical care, the synthesis will be by level of health facility/hospital, health services offered, type of costs reported, data sources, study designs, geographical location (rural and urban). The forms of critical care will be synthesized by level of health facility. The rationale for this analysis is that cost and forms of critical care vary between the subgroups mentioned above.	Page 7	
**2** Describe the standardised metric and transformation methods used	The costs will be presented in two currencies that is, the USD and TZS. All extracted costs will be adjusted to the 2019 value of USD and TZS using the World Bank GDP deflators. The GDP deflator method is explained by Chowdhury[1].	Page 7	
**3** Describe the synthesis methods	Collection and reported of cost data are usually heterogenous making it difficult to have reliable and representative findings after pooling the reported costs from different papers. In order to synthesize results in this study, we plan to categorize costs according to source of the costs, type of costs reported and costs per services offered. This will give us a summative understanding of the results.<br><br>For forms of critical care, we are planning to synthesize this data by categorizing it by level of health facility/hospital because different levels of health facilities have different amount of resources to deliver critical care thereby delivering varied critical care services.	Page 7	
**4** Criteria used to prioritise	The criteria used to prioritise results will be based on the objectives that this study sets up to achieve which rotate around forms of critical care and cost of critical care which includes resources	Page 5 and Appendix 1	

For peer review only - http://bmjopen.bmj.com/site/about/guidelines.xhtml

results for summary and synthesis	used. Studies will be included in this study using the inclusion and exclusion criteria available in the appendix.		
**5** Investigation of heterogeneity in reported effects	Heterogeneity of the costs will be investigated using the appraisal checklist.	Page 7	
**6** Certainty of evidence	In order to assess certainty of evidence, we plan to use the Reference Case for Estimating the Costs of Global Health Services and Interventions[25] to identify likely bias in the reported findings of included studies. In addition, for studies that do not report costs, we will use the Newcastle Ottawa Scale to assess bias. Any study that scores less than 5 will be excluded.	Page 7	
**7** Data presentation methods	We will use tables to describe the study characteristics and costs. The table of characteristics will contain the details of studies like author, year of publication, study design, intervention, health facility level among others). The costs will be presented in different tables by category of the cost e.g equipment, services, human resources, diagnosis.<br><br>In addition, the forms of critical care will be presented using a figure showing the different formats of critical care at the different levels of the health system.	Page 7	
Results			
**8** Reporting results	N/A (This will be reported on after the data extraction is done)	N/A	
Discussion			
**9** Limitations of the synthesis	N/A (This will be responded to after the synthesis is done)	N/A	

For peer review only - http://bmjopen.bmj.com/site/about/guidelines.xhtml

Source: Campbell M, McKenzie JE, Sowden A, Katikireddi SV, Brennan SE, Ellis S, Hartmann-Boyce J, Ryan R, Shepperd S, Thomas J, Welch V, Thomson H. Synthesis without meta-analysis (SWiM) in systematic reviews: reporting guideline BMJ 2020;368:l6890 http://dx.doi.org/10.1136/bmj.l6890

For peer review only

BMJ Open

Resource availability, utilization and cost in the provision of critical care in Tanzania: A protocol for a systematic review

Journal:	*BMJ Open*
Manuscript ID	bmjopen-2021-050881.R2
Article Type:	Protocol
Date Submitted by the Author:	09-Aug-2021
Complete List of Authors:	Kazibwe, Joseph; Imperial College London, Department of Infectious Disease Epidemiology<br>Shah, Hiral A.; Imperial College London, Department of Infectious Disease Epidemiology; Center for Global Development, Department of Infectious Disease Epidemiology<br>Kuwawenaruwa, A; Ifakara Health Institute<br>Schell, Carl Otto; Karolinska Institutet, Department of Global Public Health; Uppsala University, Department of Global Public Health<br>Khalid, Karima; Ifakara Health Institute; Muhimbili University of Health and Allied Sciences<br>Tran, Phuong Bich; University of Antwerp, Department of Family and Population Health<br>Ghosh, Srobana; Center for Global Development<br>Baker, Tim; Karolinska Institutet, Department of Global Public Health<br>GUINNESS, LORNA; Center for Global Development; London School of Hygiene and Tropical Medicine, GLOBAL HEALTH & DEVELOPMENT
**Primary Subject Heading**:	Emergency medicine
Secondary Subject Heading:	Cardiovascular medicine, Communication
Keywords:	INTENSIVE & CRITICAL CARE, HEALTH ECONOMICS, Health policy < HEALTH SERVICES ADMINISTRATION & MANAGEMENT

SCHOLARONE™ Manuscripts

I, the Submitting Author has the right to grant and does grant on behalf of all authors of the Work (as defined in the below author licence), an exclusive licence and/or a non-exclusive licence for contributions from authors who are: i) UK Crown employees; ii) where BMJ has agreed a CC-BY licence shall apply, and/or iii) in accordance with the terms applicable for US Federal Government officers or employees acting as part of their official duties; on a worldwide, perpetual, irrevocable, royalty-free basis to BMJ Publishing Group Ltd ("BMJ") its licensees and where the relevant Journal is co-owned by BMJ to the co-owners of the Journal, to publish the Work in this journal and any other BMJ products and to exploit all rights, as set out in our licence.

The Submitting Author accepts and understands that any supply made under these terms is made by BMJ to the Submitting Author unless you are acting as an employee on behalf of your employer or a postgraduate student of an affiliated institution which is paying any applicable article publishing charge ("APC") for Open Access articles. Where the Submitting Author wishes to make the Work available on an Open Access basis (and intends to pay the relevant APC), the terms of reuse of such Open Access shall be governed by a Creative Commons licence – details of these licences and which Creative Commons licence will apply to this Work are set out in our licence referred to above.

Other than as permitted in any relevant BMJ Author's Self Archiving Policies, I confirm this Work has not been accepted for publication elsewhere, is not being considered for publication elsewhere and does not duplicate material already published. I confirm all authors consent to publication of this Work and authorise the granting of this licence.

For peer review only

Resource availability, utilization and cost in the provision of critical care in Tanzania: A protocol for a systematic review

For peer review only

Corresponding Author

Joseph Kazibwe
Department of Infectious Disease Epidemiology
Imperial College London, London
Norfolk Place, London W2 1PG – UK
j.kazibwe@imperial.ac.uk
+447561876477

Authors and affiliation

Joseph Kazibwe[1,9], Hiral A. Shah[1,5], August Kuwawenaruwa[2], Carl Otto Schell[7,8], Karima Khalid[2,3], Phuong Bich Tran[4], Srobana Ghosh[5], Tim Baker[2,6], Lorna Guinness[5]

1. Department of Infectious Disease Epidemiology, Imperial College London, London – UK
2. Ifakara Health Institute, Dar es Salaam – Tanzania
3. Muhimbili University of Health and Allied Sciences, Dar es Salaam – Tanzania
4. Department of Family and Population Health (FAMPOP), University of Antwerp, Antwerp - Belgium
5. Center for Global Development (CGD), London – UK
6. Department of Clinical Research, London School of Hygiene and Tropical Medicine, UK
7. Department of Global Public Health, Karolinska Institutet, Stockholm, Sweden
8. Centre for Clinical Research Sörmland, Uppsala University, Eskilstuna, Sweden
9. Department of Global Health and Development, London School of Hygiene and Tropical Medicine, UK

Word count (excluding title page, abstract, references, figures and tables): 2,043

Key words: Critical care, costing, utilization of resources in critical care, forms of critical care, critical illness, low resource settings

For peer review only - http://bmjopen.bmj.com/site/about/guidelines.xhtml

Abstract

Introduction

Critical care is essential in saving lives of those that are critically ill, however, provision of critical care can be costly and heterogenous across lower resource settings. This paper describes the protocol for a systematic review of the literature that aims to identify the reported costs and resources available for the provision of critical care and the forms of critical care provision in Tanzania.

Methods and analysis

The review will follow the Preferred Reporting Items for Systematic Reviews and Meta-Analyses (PRISMA) guidelines. Three databases (MEDLINE, Embase and Global Health) will be searched to identify articles that report the forms of critical care, resources used in the provision of critical care in Tanzania, their availability and the associated costs. The search strategy will be developed from four key concepts; critical care provision, critical illness, resource use, Tanzania. The articles that fulfil the inclusion and exclusion criteria will be assessed for quality using the Reference Case for Estimating the Costs of Global Health Services and Interventions checklist. The extracted data will be summarized using descriptive statistics including frequencies, mean and median of the quantity and costs of resources used in the components of critical care services, depending on the data availability. This study will be carried out between February to November 2021.

Ethics and dissemination

This study is a review of secondary data and ethical clearance was sought from and granted by the Tanzanian National Institute of Medical Research (reference: NIMR/HQ/R.8a/Vol. IX/3537) and London School of Hygiene and Tropical Medicine (LSHTM ethics ref: 22866). We will publish the review in a peer reviewed journal as an open access article in addition to presenting the findings at conferences and public scientific gatherings.

Strengths and weaknesses of the proposed study

- This study will follow the PRISMA guidelines thereby being the first systematic review of the literature around the costs and resources used in critical care and forms of critical care in a lower resource setting such as Tanzania.
- The search strategy broadly and comprehensively includes studies on critical care and costs of inpatient care in Tanzania increasing chances of including all published studies on the subject.
- This study has a fully developed population, intervention, comparison and outcome (PICO) framework.
- The study will include only articles published in English.
- There is a chance that there may be limited relevant data on Tanzania in that there may also be a publication bias where published literature focuses on specific diseases (e.g. malaria, pneumonia).

For peer review only

Background

The COVID19 pandemic is now synonymous with being a critical care crisis with both high income and low-and-middle income countries (LMICs) struggling to meet demand for critical care services.[1,2] This has led to the care of critical illness becoming an urgent issue and a point of focus in global health with stakeholders providing recommendations on what interventions should be scaled up, while knowledge on the potential costs involved remains sparse[1,3,4].

Critical care entails the care given to a patient in need of specialist monitoring, treatment and attention, for example for a patient with a life-threatening illness or injury[5]. Over the years, there have been great advances in the provision of critical care for example invasive and non-invasive monitoring techniques, mechanical ventilation (MV), and renal replacement therapy, among others which have resulted in reduced mortality rates over time among patients with critical illness especially in high income countries[6].

Despite the advances made in the provision of critical care in LMICs[7–9], the limited availability of intensive care units (ICUs) and the low numbers of trained personnel in such settings persist. In addition, the frequent power cuts and inadequate supply of oxygen in some areas make advanced care challenging. The equipment in an ICU can be expensive and may not be affordable or available in many LMICs with an already constrained healthcare budget[10,11]. In a global mapping of ICU bed capacity, the ICU bed density in the majority of Sub-Saharan African countries was reported to be below 1.0 ICU bed per 100,000 population as compared with more than 25 in Germany and the USA[12]. Even where ICUs are available in LMICs settings, the services offered are expensive, making them inaccessible and unaffordable for the majority of critically ill patients. This limited availability of critical care services in LMICs has been linked to the high ICU related deaths of between 30%-80% of all ICU admissions[8]. In fact, most critically ill patients are cared for in general hospital wards[13,14].

Given the constraints that LMICs face when having to provide critical care, recent research has proposed the concept of Essential Emergency and Critical Care (EECC) which focuses on providing low-cost care for critical illness in any place within the hospital[1,9]. EECC consists of effective life-saving actions of low cost and complexity, is appropriate for all hospital settings and for all critically ill patients irrespective of their age, sex, location or underlying medical condition[9]. EECC has been proposed as a key component in universal health coverage (UHC). Research is ongoing into the policy and economic aspects of EECC in Tanzania and Kenya to inform EECC implementation in these and other low resourced health system settings.

To inform this work, it is necessary to review the existing knowledge about the current resource availability, utilization and cost in the provision of critical care in Tanzania. Several studies have been conducted to estimate the resource use and cost of critical care globally but the majority of these estimates are confined to high income countries and may not be generalizable to the LMICs settings [15–17]. Even studies that look at the resource use and cost of critical care, whose findings are reported as international estimates, focus on high income countries for example Germany, Hungary, UK and France[18]. In addition, the majority of studies focus on the costs incurred in an ICU only[17] which may not reflect the resources used for all critically illness, especially in LMICs. Here, we describe the protocol of a systematic review that will identify the existing evidence on the quantification of current resources available and the economic costs in the provision of critical care in Tanzania.

For peer review only - http://bmjopen.bmj.com/site/about/guidelines.xhtml

Aim

This paper describes the protocol for a systematic review of the literature that aims to identify the reported costs and resources available for the provision of critical care and the forms of critical care provision in Tanzania.

Research questions

1. What are forms of critical care provided in the health system in Tanzania?
2. What resources are reported to be available and have been utilised in the provision of critical care in Tanzania?
3. What are the reported costs of available resources (including oxygen supplies) used in critical care provision in Tanzania?

Methods

The systematic review will follow the Preferred Reporting Items for Systematic Reviews and Meta-Analyses (PRISMA) guidelines[19] and the protocol was registered with PROSPERO; registration number: CRD42020221923.

The review will use a PICO (Population, Intervention, Comparator, Outcome) framework to enable a comprehensive and unbiased search of existing literature and identify the key search terms[20] (see table 1) . Critical care as a concept does not have an internationally accepted definition. The review defines critical care as the components of care that support a critically ill patient's vital organ functions where critical illness is a state of ill health with vital organ dysfunction, a high risk of imminent death if action is not taken and the potential for reversibility. A critically ill patient is one with acute need for life saving organ support. Critical care is ideally team-based and multi-professional and can be given in any location. Critical care can be high-intensity e.g mechanical ventilation, requiring highly specialised services, or it can be low intensity resuscitative and supportive care e.g oxygen provided using a face mask.

Table 1: PICO (Population, Intervention, Comparator, Outcome) Framework used to inform the search strategy.

**Element**	**Description**
Population	Any patient in need of critical care
Intervention	Inpatient services that could form part of critical care provision
Comparison	No critical care
Outcomes	Critical care services available<br>Cost of the different inputs to critical care provision per patient<br>Quantity and type of resources used in provision of critical care per patient

For peer review only - http://bmjopen.bmj.com/site/about/guidelines.xhtml

Definitions

Forms of critical care are the different levels of critical care services (according to level of advancement) that can be offered to a critically ill patient that can range from basic services like oxygen therapy in general wards to mechanical ventilation in ICUs.

Resources (in this study) are the physical items, material or equipment used in the provision of critical care in a given setting. Resources will be classified using a standard of classification, that is; by input (human resources, consumables etc) or by activities (diagnostics, bed days etc) depending on the data available.

Search strategy

Electronic databases (Medline (Ovid), Embase (embase.com) and Global Health) will be systematically searched for articles published between 2010 to present. A ten-year time frame will be considered to ensure that findings reflect the present-day resource utilization paradigm in critical care provision as resources used and their costs vary with the nature of technology and clinical guidelines in place. Bibliographies of included articles will be reviewed to find relevant articles that fulfil the inclusion criteria but not yet identified. Authors whose abstracts will be considered for inclusion, but the full text is not accessible online, will be contacted and requested for article access. Google will also be used to search for published articles that may not have been indexed within the databases. As internet search engines typically return several thousand results, the searches will be restricted to the first fifty hits and links to potentially relevant material will be accessed[21]. The search will be done in anonymous mode to ensure that we do not pick up searcher's embedded preferences. In addition, the involvement of Tanzanian experts in health economics and critical care will be part of the study team to ensure that no obvious articles or reports are missed.

Due to the complex nature of critical care, a combination of strategies was used to identify key search terms that can capture critical illness and critical care. First, the core concepts that we will search on were identified through the PICO framework: critical care (inpatient services that constitute critical care provision), critical illness, resource use (resource utilisation and cost), setting (Tanzania). Subsequently, to identify the search terms, we reviewed the MESH terms related to the key concepts in Medline, consulted within our expert group and scoped our existing critical care and costing libraries. We also include leading causes of death in Tanzania in the search string (e.g. neonatal disorders, HIV, Malaria and tuberculosis (TB)[22]), on the premise that these are conditions that have a high risk of critical illness. Variations of the key words will be included in the search string. The concepts and search terms are listed in table 2. Search strategies were tailored to search the three databases. Search strategies for each database are available in appendix 1.

Table 2: Key concepts and search terms identified for the search.

Concept	Search terms
Inpatient services that can be considered to form part of critical care provision	***Critical care:*** Critical care, emergency care, critical emergency care, essential emergency and critical care, intensive care, essential critical care, early goal directed therapy, neonatal, acute care, emergency medicine, trauma,

	emergency medical services, ICU, Peri-operative care, maternal emergencies, cardiovascular/inotropic support, renal support<br>***General inpatient care:***<br>General ward care, general inpatient care, general care, hospital care<br>***Oxygen therapy:***<br>Oxygen therapy/provision, artificial ventilation, mechanical ventilation Non-invasive ventilation (NIV), Continuous Positive Airway Pressure (CPAP), High flow (nasal) oxygen (HFO, HFNO)
Critical illness	***Critical illness:***<br>Critical illness, sick children, acute paediatrics, emergency obstetric care, poly-trauma, Severe illness, Life-threatening illness, Acute illness, Emergency illness<br>***Leading causes of mortality in Tanzania:***<br>HIV, Malaria, TB, sepsis, trauma, burns, pneumonia, emergency surgery, shock, haemorrhage, respiratory failure, coma, unconsciousness, meningitis, choking, anuria, acute kidney injury
Resource use	Cost, expen-, spending, invest-<br>Financ-, financial burden, financial impact, financial consequence, economic, economic burden, economic impact, economic consequence<br>Direct cost, indirect cost, medical cost, non-medical cost, nonmedical cost, opportunity cost<br>Resource, resource use, resource utilisation, resource utilization, health service use, health service utilisation, health service utilization<br>Provider cost, health system cost, hospital cost, system cost, provider cost, hospital cost, societal cost, insurance, reimburse-, cost of illness, cost analysis, economic modelling
Tanzania	Tanzania

Eligibility criteria

A study will be considered eligible if it is published in English and reports on forms/types of critical care, services offered under critical care, resources and or costs incurred by the health system or health providers in providing critical care in Tanzania. A detailed inclusion exclusion criterion can be found in appendix 2.

For peer review only

Study Selection
The PRISMA guidelines[23] will be followed in the selection process. The number of articles retrieved will be listed and uploaded to Rayyan QCRI software which will be used to identify and remove duplicates[24]. Eligibility of identified studies will be assessed independently by researchers LG, HS, SG, PT and JK by first reviewing the title and abstract. All conflicts will be discussed and agreed upon through consensus with third researcher (LG or HS) when applicable. The eligibility of each of the remaining studies will be decided on through full text review of each paper by at least two researchers (SG, JK, HS, PT, LG).

Data extraction process
A data extraction form including author, year of publication, context (location, setting- urban or rural, type of facility, level of facility), critical care services offered (including special critical care services), critical care equipment available, costing perspective, costing year, currency used, type of provider, payer, source of cost data, costing time frame, direct medical costs, resources used, cost ingredients will be developed.

Simultaneously during the data extraction process, studies will be assessed for similarity in cost ingredients to be included in the quantitative (cost) synthesis.

Risk of bias in individual studies
The quality of the included articles will be assessed using the Reference Case for Estimating the Costs of Global Health Services and Interventions[25], which provides a framework for quality assessment and data extraction. While standard practice for review of economic evaluations is the Consolidated Health Economic Evaluation Reporting Standards (CHEERS) tool, this does not provide sufficient information to assess the quality of cost studies. Any discrepancies will be addressed by a joint re-evaluation of the article among all authors. The appraisal checklist[26] will be used to assess the similarity between the reported costs. For studies that do not report costs, the Newcastle Ottawa Scale will be used to assess their quality[27].

Summary measures and synthesis of results
Studies will be grouped into two; 1) those that report on costs and 2) those that report on forms of critical care. The summary measures used in this study will be descriptive statistics such as frequencies, mean and median critical care services, costs and quantities identified during the data extraction. Confidence intervals of the point estimates of costs and resource quantities will be captured when reported by the included studies. Group analysis of the results will be done along level of health facility/hospital, health services offered, cost reported, data sources, study designs, geographical location (rural and urban).

Synthesis of the costs and resources will be performed. Costs, average costs/resources per patient of the different components of critical care will be estimated and converted to 2019 USD and TZS, using the World Bank GDP deflators[28]. The costs will be presented per the different components of critical care. A synthesis without meta-analysis (SWiM) checklist[29] will be used to guide the synthesis. A detailed checklist is available as an appendix (appendix 3). The PRISMA checklist has been included too (appendix 4).

Timeline

The study is expected to be completed by 31st November 2021.

Patient and public involvement

Patients and the public will not be involved in this research, as this study will focus on reviewing secondary publicly accessible data.

Ethics and Dissemination

For peer review only - http://bmjopen.bmj.com/site/about/guidelines.xhtml

This study is a review of secondary data, and ethical clearance was sought from and granted by The Tanzanian National Institute of Medical Research (reference: NIMR/HQ/R.8a/Vol. IX/3537) and London School of Hygiene and Tropical Medicine (LSHTM ethics ref: 22866).

Following completion of the systematic review, we shall assess whether the research aim and questions have been met and answered respectively using the Grading of Recommendations, Assessment, Development and Evaluations frame work[30].

We aim to publish our findings in a peer reviewed journal as an open access article in addition to presenting the findings at conferences and public scientific gatherings. These findings will also be a basis and provide special input for a cost effectiveness study of EECC in Tanzania.

For peer review only

References

1. Baker T, Otto Schell C, Brun Petersen D, et al. Essential care of critical illness must not be forgotten in the COVID-19 pandemic. *Lancet*. 2020;395:1253-1254. doi:10.1016/S0140-6736(20)30793-5
2. Barasa E, Kairu A, Nganga W, et al. Examining Unit Costs for COVID-19 Case Management in Kenya. *medRxiv*. Published online October 13, 2020:2020.10.08.20209684. doi:10.1101/2020.10.08.20209684
3. World Bank. World Bank facilitates immediate support and resources for the COVID-19 (Coronavirus) emergency in Bolivia. Published April 2, 2020. Accessed December 16, 2020. https://www.worldbank.org/en/news/press-release/2020/04/02/el-banco-mundial-facilita-apoyo-y-recursos-inmediatos-para-atender-la-emergencia-por-el-covid-19-coronavirus-en-bolivia
4. The World Bank. *The World Bank Annual Report 2020: Supporting Countries in Unprecedented Time*.; 2020. Accessed December 16, 2020. https://www.worldbank.org/en/about/annual-report/covid-response
5. National Institute for Health and Care Excellence (NICE). What "critical care" means | Information for the public | Rehabilitation after critical illness in adults | Guidance | NICE. Published March 25, 2009. Accessed November 1, 2020. https://www.nice.org.uk/guidance/cg83/ifp/chapter/What-critical-care-means
6. Zimmerman JE, Kramer AA, Knaus WA. Changes in hospital mortality for United States intensive care unit admissions from 1988 to 2012. *Crit Care*. 2013;17(2):R81. doi:10.1186/cc12695
7. Murthy S, Adhikari NK. ATS REPORTS Global Health Care of the Critically Ill in Low-Resource Settings. *Ann Am Thorac Soc*. 2013;10(5):509-513. doi:10.1513/AnnalsATS.201307-246OT
8. Vukoja M, Riviello ED, Schultz MJ. Critical care outcomes in resource-limited settings. *Curr Opin Crit Care*. 2018;24(5):421-427. doi:10.1097/MCC.0000000000000528
9. Schell CO, Gerdin Wärnberg M, Hvarfner A, et al. The global need for essential emergency and critical care. *Crit Care*. 2018;22(1):1-5. doi:10.1186/s13054-018-2219-2
10. Turner HC, Van Hao N, Yacoub S, et al. Achieving affordable critical care in low-income and middle-income countries Commentary Handling editor Seye Abimbola. *BMJ Glob Heal*. 2019;4:1675. doi:10.1136/bmjgh-2019-001675

For peer review only - http://bmjopen.bmj.com/site/about/guidelines.xhtml

11. Adhikari NKJ, Fowler RA, Bhagwanjee S, Rubenfeld GD. Critical care and the global burden of critical illness in adults. *Lancet*. 2010;376(9749):1339-1346. doi:10.1016/S0140-6736(10)60446-1

12. Ma X, Vervoort D. Critical care capacity during the COVID-19 pandemic: Global availability of intensive care beds. *J Crit Care*. 2020;58(January):96-97. http://europepmc.org/backend/ptpmcrender.fcgi?accid=PMC7194590&blobtype=pdf

13. Dart PJ, Kinnear J, Bould MD, Mwansa SL, Rakhda Z, Snell D. An evaluation of inpatient morbidity and critical care provision in Zambia. *Anaesthesia*. 2017;72(2):172-180. doi:10.1111/anae.13709

14. Kayambankadzanja RK, Schell CO, Namboya F, et al. The prevalence and outcomes of sepsis in adult patients in two hospitals in Malawi. *Am J Trop Med Hyg*. 2020;102(4):896-901. doi:10.4269/ajtmh.19-0320

15. Lone NI, Seretny M, Wild SH, Rowan KM, Murray GD, Walsh TS. Surviving intensive care: A systematic review of healthcare resource use after hospital discharge. *Crit Care Med*. 2013;41(8):1832-1843. doi:10.1097/CCM.0b013e31828a409c

16. Abu Al-Saad N, Skedgel C, Nortje J. Principles of resource allocation in critical care. *BJA Educ*. 2017;17(12):390-395. doi:10.1093/bjaed/mkx029

17. Woo BFY, Lee JXY, Tam WWS. The impact of the advanced practice nursing role on quality of care, clinical outcomes, patient satisfaction, and cost in the emergency and critical care settings: A systematic review. *Hum Resour Health*. 2017;15(1):1-22. doi:10.1186/s12960-017-0237-9

18. Negrini D, Sheppard L, Mills GH, et al. International Programme for Resource Use in Critical Care (IPOC) - A methodology and initial results of cost and provision in four European countries. *Acta Anaesthesiol Scand*. 2006;50(1):72-79. doi:10.1111/j.1399-6576.2006.00901.x

19. Shamseer L, Moher D, Clarke M, et al. Preferred reporting items for systematic review and meta-analysis protocols (prisma-p) 2015: Elaboration and explanation. *BMJ*. 2015;349(January):1-25. doi:10.1136/bmj.g7647

20. Higgins J, Thomas J, Chandler J, et al. *Cochrane Handbook for Systematic Reviews of Interventions*. Cochrane; 2020. https://training.cochrane.org/handbook/current

21. Savilaakso S, Garcia C, Garcia-Ulloa J, et al. Systematic review of effects on biodiversity from oil palm production. *Environ Evid*. 2014;3(1):4. doi:10.1186/2047-2382-3-4

22. Abbafati C, Machado DB, Cislaghi B, et al. Global burden of 369 diseases and injuries in 204 countries and territories, 1990–2019: a systematic analysis for the Global Burden of Disease Study 2019. *Lancet*. 2020;396(10258):1204-1222. doi:10.1016/S0140-6736(20)30925-9

23. Moher D, Liberati A, Tetzlaff J, et al. Preferred reporting items for systematic reviews and meta-analyses: The PRISMA statement (Chinese edition). *J Chinese Integr Med*. 2009;7(9):889-896. doi:10.3736/jcim20090918

24. Ouzzani M, Hammady H, Fedorowicz Z, Elmagarmid A. Rayyan-a web and mobile app for systematic reviews. *Syst Rev*. 2016;5(1). doi:10.1186/s13643-016-0384-4

25. Vassall A, Sweeney S, Kahn JG, et al. *Reference Case for Estimating the Costs of Global Health Services and Interventions*.

26. Adawiyah R al, Saweri OPM, Boettiger DC, et al. The costs of scaling up HIV and syphilis testing in

For peer review only

low- and middle-income countries: a systematic review. *Health Policy Plan*. 2021;2021:1-16. doi:10.1093/heapol/czab030

27. Wells G, Shea B, O'Connell D, et al. The Newcastle-Ottawa Scale (NOS) for assessing the quality of nonrandomised studies in meta-analyses. Ottawa Hospital Research Institute . Published 2019. Accessed February 23, 2021. http://www.ohri.ca/programs/clinical_epidemiology/oxford.asp

28. World bank. GDP deflator (base year varies by country) - Tanzania . Accessed June 2, 2021. https://data.worldbank.org/indicator/NY.GDP.DEFL.ZS?locations=TZ

29. Campbell M, McKenzie JE, Sowden A, et al. Synthesis without meta-analysis (SWiM) in systematic reviews: Reporting guideline. *BMJ*. 2020;368. doi:10.1136/bmj.l6890

30. Guyatt G, Oxman AD, Akl EA, et al. GRADE guidelines: 1. Introduction - GRADE evidence profiles and summary of findings tables. *J Clin Epidemiol*. 2011;64(4):383-394. doi:10.1016/j.jclinepi.2010.04.026

For peer review only

Authors' contributions

All authors made sufficient contribution to the development of manuscript in line with ICMJE criteria. Below is the detailed breakdown of their contribution.

Concept and design: JK, HS, LG, PT

Development of first draft: JK

Acquisition of data: N/A

Analysis and interpretation of data: N/A

Drafting of the manuscript: JK, LG, HS, SG, PT, COS, KK and AK

Critical revision of paper for important intellectual content: LG, HS, SG, PT, TB, COS, KK and AK

Statistical analysis: N/A

Provision of study materials: JK, TB, LG, PT, COS, KK and AK

Obtaining funding: TB, COS

Administrative and technical support: JK, LG, HS, SG, PT, TB, COS, KK and AK

Supervision: LG, HS

Funding statement

This work was supported by the Wellcome Trust [221571/Z/20/Z], as part of the 'Innovation in low-and middle-income countries' Flagship.

Competing interests statement

Authors declare no competing interests and the funders did not in any way influence the development of the development of this protocol.

For peer review only - http://bmjopen.bmj.com/site/about/guidelines.xhtml

Appendix 1: Search Strategies

1) Medline search strategy

	Interface: Ovid MEDLINE<br>Date of Search:<br>Number of hits:
**Number**	**Search**
1	(((Critical care or emergency care or critical emergency care or essential emergency) and critical care) or intensive care or early goal directed therapy or neonatal or acute care or emergency medicine or trauma or emergency medical services or ICU or Peri-operative care or maternal emergencies or cardiovascular support or inotropic support or renal support).mp.
2	(General ward care or general inpatient care or hospital care or Oxygen therapy or artificial ventilation or mechanical ventilation or Non-invasive ventilation or Continuous Positive Airway Pressure or High flow nasal oxygen or HFO or HFNO).mp.
3	(Critical illness or sick children or acute paediatrics or emergency obstetric care or poly-trauma or Severe illness or Life-threatening illness or Acute illness or HIV or Malaria or TB or sepsis or trauma or burns or pneumonia or emergency surgery or shock or haemorrhage or respiratory failure or coma or unconsciousness or meningitis or choking or anuria or acute kidney injury).mp.
4	(Cost or expen or spending or invest- or Financ or financial burden or financial impact or financial consequence or economic or economic burden or economic impact or economic consequence or Direct cost or indirect cost or medical cost or non-medical cost or nonmedical cost or opportunity cost or Resource or resource or resource utilisation or resource utilization or health service utilisation or health service utilization or Provider cost or health system cost or hospital cost or system cost or provider cost or hospital cost or societal cost or insurance or reimburse- or cost of illness or cost analysis or economic modelling).mp.
5	Tanzania.mp. or exp Tanzania/
6	1 or 2 or 3 or 4
7	5 and 6
8	limit 7 to yr="2010 -Current"

2) Embase search strategy

	Interface: Ovid Embase<br>Date of Search:

For peer review only

	Number of hits:
**Number**	**Search**
1	(((Critical care or emergency care or critical emergency care or essential emergency) and critical care) or intensive care or early goal directed therapy or neonatal or acute care or emergency medicine or trauma or emergency medical services or ICU or Peri-operative care or maternal emergencies or cardiovascular support or inotropic support or renal support).mp.
2	(General ward care or general inpatient care or hospital care or Oxygen therapy or artificial ventilation or mechanical ventilation or Non-invasive ventilation or Continuous Positive Airway Pressure or High flow nasal oxygen or HFO or HFNO).mp.
3	(Critical illness or sick children or acute paediatrics or emergency obstetric care or poly-trauma or Severe illness or Life-threatening illness or Acute illness or HIV or Malaria or TB or sepsis or trauma or burns or pneumonia or emergency surgery or shock or haemorrhage or respiratory failure or coma or unconsciousness or meningitis or choking or anuria or acute kidney injury).mp.
4	(Cost or expen or spending or invest- or Financ or financial burden or financial impact or financial consequence or economic or economic burden or economic impact or economic consequence or Direct cost or indirect cost or medical cost or non-medical cost or nonmedical cost or opportunity cost or Resource or resource or resource utilisation or resource utilization or health service utilisation or health service utilization or Provider cost or health system cost or hospital cost or system cost or provider cost or hospital cost or societal cost or insurance or reimburse- or cost of illness or cost analysis or economic modelling).mp.
5	Tanzania.mp. or exp Tanzania/
6	1 or 2 or 3 or 4
7	5 and 6
8	limit 7 to yr="2010 -Current"

For peer review only

3) Global health Search Strategy

	Interface: Ovid Global Health<br>Date of Search:<br>Number of hits:
**Number**	**Search**
1	(((Critical care or emergency care or critical emergency care or essential emergency) and critical care) or intensive care or early goal directed therapy or neonatal or acute care or emergency medicine or trauma or emergency medical services or ICU or Peri-operative care or maternal emergencies or cardiovascular support or inotropic support or renal support).mp.

2	(General ward care or general inpatient care or hospital care or Oxygen therapy or artificial ventilation or mechanical ventilation or Non-invasive ventilation or Continuous Positive Airway Pressure or High flow nasal oxygen or HFO or HFNO).mp.
3	(Critical illness or sick children or acute paediatrics or emergency obstetric care or poly-trauma or Severe illness or Life-threatening illness or Acute illness or HIV or Malaria or TB or sepsis or trauma or burns or pneumonia or emergency surgery or shock or haemorrhage or respiratory failure or coma or unconsciousness or meningitis or choking or anuria or acute kidney injury).mp.
4	(Cost or expen or spending or invest- or Financ or financial burden or financial impact or financial consequence or economic or economic burden or economic impact or economic consequence or Direct cost or indirect cost or medical cost or non-medical cost or nonmedical cost or opportunity cost or Resource or resource or resource utilisation or resource utilization or health service utilisation or health service utilization or Provider cost or health system cost or hospital cost or system cost or provider cost or hospital cost or societal cost or insurance or reimburse- or cost of illness or cost analysis or economic modelling).mp.
5	Tanzania.mp. or exp Tanzania/
6	1 or 2 or 3 or 4
7	5 and 6
8	limit 7 to yr="2010 -Current"

For peer review only

Appendix 2: Inclusion and exclusion criteria

Inclusion criterion

For a study to be included it should fulfil the following criterion

- Published in English in a peer reviewed journal
- Reports forms or types of critical care offered, critical care services offered and or costs and resources used in the provision of care in Tanzania
- Includes costs from a provider perspective
- Articles published from 2010

Exclusion criterion

Studies will be excluded in case of the following;

- Published in any other languages except English
- Studies carried out outside Tanzania
- Studies with costs estimated from patient perspective
- Studies without full text versions
- Studies published before 2010

For peer review only

Synthesis Without Meta-analysis (SWiM) reporting items

SWiM is intended to complement and be used as an extension to PRISMA			
**SWiM reporting item**	**Item description**	**Page in manuscript where item is reported**	**Other***
Methods			
**1** Grouping studies for synthesis	The studies will be grouped into two; studies that report cost and studies that report on forms of critical care. For the studies that report costs of critical care, the synthesis will be by level of health facility/hospital, health services offered, type of costs reported, data sources, study designs, geographical location (rural and urban). The forms of critical care will be synthesized by level of health facility. The rationale for this analysis is that cost and forms of critical care vary between the subgroups mentioned above.	Page 7	
**2** Describe the standardised metric and transformation methods used	The costs will be presented in two currencies that is, the USD and TZS. All extracted costs will be adjusted to the 2019 value of USD and TZS using the World Bank GDP deflators. The GDP deflator method is explained by Chowdhury[1].	Page 7	
**3** Describe the synthesis methods	Collection and reported of cost data are usually heterogenous making it difficult to have reliable and representative findings after pooling the reported costs from different papers. In order to synthesize results in this study, we plan to categorize costs according to source of the costs, type of costs reported and costs per services offered. This will give us a summative understanding of the results.<br><br>For forms of critical care, we are planning to synthesize this data by categorizing it by level of health facility/hospital because different levels of health facilities have different amount of resources to deliver critical care thereby delivering varied critical care services.	Page 7	
**4** Criteria used to prioritise	The criteria used to prioritise results will be based on the objectives that this study sets up to achieve which rotate around forms of critical care and cost of critical care which includes resources	Page 5 and Appendix 1	

For peer review only - http://bmjopen.bmj.com/site/about/guidelines.xhtml

results for summary and synthesis	used. Studies will be included in this study using the inclusion and exclusion criteria available in the appendix.		
**5** Investigation of heterogeneity in reported effects	Heterogeneity of the costs will be investigated using the appraisal checklist.	Page 7	
**6** Certainty of evidence	In order to assess certainty of evidence, we plan to use the Reference Case for Estimating the Costs of Global Health Services and Interventions[25] to identify likely bias in the reported findings of included studies. In addition, for studies that do not report costs, we will use the Newcastle Ottawa Scale to assess bias. Any study that scores less than 5 will be excluded.	Page 7	
**7** Data presentation methods	We will use tables to describe the study characteristics and costs. The table of characteristics will contain the details of studies like author, year of publication, study design, intervention, health facility level among others). The costs will be presented in different tables by category of the cost e.g equipment, services, human resources, diagnosis.<br><br>In addition, the forms of critical care will be presented using a figure showing the different formats of critical care at the different levels of the health system.	Page 7	
Results			
**8** Reporting results	N/A (This will be reported on after the data extraction is done)	N/A	
Discussion			
**9** Limitations of the synthesis	N/A (This will be responded to after the synthesis is done)	N/A	

For peer review only - http://bmjopen.bmj.com/site/about/guidelines.xhtml

Source: Campbell M, McKenzie JE, Sowden A, Katikireddi SV, Brennan SE, Ellis S, Hartmann-Boyce J, Ryan R, Shepperd S, Thomas J, Welch V, Thomson H. Synthesis without meta-analysis (SWiM) in systematic reviews: reporting guideline BMJ 2020;368:l6890 http://dx.doi.org/10.1136/bmj.l6890

For peer review only

PRISMA CHECKLIST

Title of study: Resource availability, utilization and cost in the provision of critical care in Tanzania: A protocol for a systematic review

Item Number	Item	Description	Page and line number
1	Title	Identify the report as a systematic review	Page 1, lines 4-5
2	Abstract	Summary of the study	Page 2, Lines 39-61
3	Rationale	Describe the rationale for the review in the context of existing knowledge	Page 3, Lines 118-135
4	Objectives	Provide an explicit statement of the objective(s) or question(s) the review addresses	Page 4, Lines 137-146
5	Eligibility criteria	Specify the inclusion and exclusion criteria for the review and how studies were grouped for the syntheses	Page 6, Lines 205-209 And appendix 2
6	Information sources	Specify all databases, registers, websites, organisations, reference lists, and other sources searched or consulted to identify studies.	Page 5, Lines 175-187
7	Search strategy	Present the full search strategies for all databases, registers, and websites, including any filters and limits used	Page 5, Lines 175-199 And Appendix 1
8	Selection process	Specify the methods used to decide whether a study met the inclusion criteria of the review, including how many reviewers screened each record and each report retrieved, whether they worked independently, and, if applicable, details of automation tools used in the process	Page 7, Lines 212-217
9	Data collection process	Specify the methods used to collect data from reports, including how many reviewers collected data from each report, whether they worked independently, any processes for obtaining or confirming data from study investigators, and, if applicable, details of automation tools used in the process	Page 7, Lines 219-224
10a	Data items	List and define all outcomes for which data were sought. Specify whether all results that were compatible with each outcome domain in each study were sought (for example, for all measures, time	Table 1: Page 4

For peer review

		points, analyses), and, if not, the methods used to decide which results to collect	
10b	Data items	List and define all other variables for which data were sought (such as participant and intervention characteristics, funding sources). Describe any assumptions made about any missing or unclear information	Page 7, Lines 219-222
11	Study risk of bias	Specify the methods used to assess risk of bias in the included studies, including details of the tool(s) used, how many reviewers assessed each study and whether they worked independently, and, if applicable, details of automation tools used in the process	Page 7, Lines 225-233
12	Effect measures	Specify for each outcome the effect measure(s) (such as risk ratio, mean difference) used in the synthesis or presentation of results	Not applicable
13a	Synthesis methods	Describe the processes used to decide which studies were eligible for each synthesis (such as tabulating the study intervention characteristics and comparing against the planned groups for each synthesis (item #5))	Page 7, Lines 222-227
13b	Synthesis methods	Describe any methods required to prepare the data for presentation or synthesis, such as handling of missing summary statistics or data conversions	Page 7, Lines 234-243
13c	Synthesis methods	Describe any methods used to tabulate or visually display results of individual studies and syntheses	Page 7, Lines 235-242
13d	Synthesis methods	Describe any methods used to synthesise results and provide a rationale for the choice(s). If meta-analysis was performed, describe the model(s), method(s) to identify the presence and extent of statistical heterogeneity, and software package(s) used	Page 7, Lines 235-242
13e	Synthesis methods	Describe any methods used to explore possible causes of heterogeneity among study results (such as subgroup analysis, meta-regression)	Not applicable
13f	Synthesis methods	Describe any sensitivity analyses conducted to assess robustness of the synthesised results	Not applicable
14	Reporting bias assessment	Describe any methods used to assess risk of bias due to missing results in a synthesis (arising from reporting biases)	Not applicable at this stage

For peer review only

15	Certainty assessment	Describe any methods used to assess certainty (or confidence) in the body of evidence for an outcome	Page 7, 237-238
16a	Study selection	Describe the results of the search and selection process, from the number of records identified in the search to the number of studies included in the review, ideally using a flow diagram	Not applicable at this stage
16b	Study selection	Cite studies that might appear to meet the inclusion criteria, but which were excluded, and explain why they were excluded	Not applicable at this stage
17	Study characteristics	Cite each included study and present its characteristics	Not applicable at this stage
18	Risk of bias in studies	Present assessments of risk of bias for each included study	Not applicable at this stage
19	Results of individual studies	For all outcomes, present for each study (a) summary statistics for each group (where appropriate) and (b) an effect estimate and its precision (such as confidence/credible interval), ideally using structured tables or plots	Not applicable at this stage
20a	Results of syntheses	For each synthesis, briefly summarise the characteristics and risk of bias among contributing studies	Not applicable at this stage
20b	Results of syntheses	Present results of all statistical syntheses conducted. If meta-analysis was done, present for each the summary estimate and its precision (such as confidence/credible interval) and measures of statistical heterogeneity. If comparing groups, describe the direction of the effect	Not applicable at this stage
20c	Results of syntheses	Present results of all investigations of possible causes of heterogeneity among study results	Not applicable at this stage
20d	Results of syntheses	Present results of all sensitivity analyses conducted to assess the robustness of the synthesised results	Not applicable at this stage
21	Risk of reporting bias in syntheses	Present assessments of risk of bias due to missing results (arising from reporting biases) for each synthesis assessed	Not applicable at this stage
22	Certainty of evidence	Present assessments of certainty (or confidence) in the body of evidence for each outcome assessed	Not applicable at this stage

For peer review only

23a	Discussion	Provide a general interpretation of the results in the context of other evidence	Not applicable at this stage
23b	Discussion	Discuss any limitations of the evidence included in the review	Not applicable at this stage
23c	Discussion	Discuss any limitations of the review processes used	Not applicable at this stage
23d	Discussion	Discuss implications of the results for practice, policy, and future research	Not applicable at this stage
24a	Registration and protocol	Provide registration information for the review, including register name and registration number, or state that the review was not registered	Page 4, Lines 150-151
24b	Registration and protocol	Indicate where the review protocol can be accessed, or state that a protocol was not prepared	Page 4, Lines 150-151
24c	Registration and protocol	Describe and explain any amendments to information provided at registration or in the protocol	Not applicable at this stage
25	Support	Describe sources of financial or non-financial support for the review, and the role of the funders or sponsors in the review	Page 10, lines 360-361
26	Competing interests	Declare any competing interests of review authors	Page 10, Lines 362-364
27	Availability of data, code and other materials	Report which of the following are publicly available and where they can be found: template data collection forms; data extracted from included studies; data used for all analyses; analytic code; any other materials used in the review	Not applicable at this stage

For peer review only
